# In Situ Modification of Sulfur-Based Cathode Electrolyte Interphases for Boosting Zinc/Graphite Dual-Ion Batteries via Vinylene Carbonate Additive and Dipropylene Glycol Methyl Ether/Water Mixed Solvent

**DOI:** 10.1021/acssuschemeng.5c01897

**Published:** 2025-05-27

**Authors:** Yitao He, Xiaoxiang Shen, Jiří Červenka

**Affiliations:** † Department of New Energy Science and Engineering, School of Energy and Environment, 47852 Anhui University of Technology, Ma’anshan 243002, China; ‡ Department of Thin Films and Nanostructures, FZU−Institute of Physics of the Czech Academy of Sciences, Cukrovarnická 10/112, 162 00 Prague 6, Czech Republic

**Keywords:** graphite cathode, zinc metal anode, cathode electrolyte interface, dipropylene glycol methyl ether, dual-ion battery

## Abstract

Dual-ion batteries (DIBs) have been extensively explored due to their low material costs, high power density, and eco-friendly characteristics. However, the graphite cathode often leads to structural damage and instability at the electrode/electrolyte interface, severely diminishing its electrochemical performance. This work presents a cost-effective approach from the perspective of electrolyte optimization to overcome these challenges. By incorporating a moderate amount (5 wt %) of vinylene carbonate (VC) as an additive into a mixed solvent of dipropylene glycol methyl ether (DPM) and water, significant improvements in electrochemical performance are achieved, primarily due to the formation of a sulfur-rich cathode electrolyte interface (CEI) on the graphite surface and the electrolyte additive fostering the generation of nanosized sulfide particles in the graphite lattice, which provide active storage sites for anions. In the graphite-Zn DIB, a high discharge-specific capacity of 140 mAh g^–1^ was achieved at 100 mA g^–1^, and after 500 cycles, the capacity retention rate is 84.2%, which is much higher than that of the battery without VC. This work demonstrates the potential of a cost-effective electrolyte in optimizing the composition of the graphite cathode CEI and promoting the formation of inorganic nanoparticle hosts on the graphite cathode surface for enhancing the performance of DIBs.

## Graphical abstract

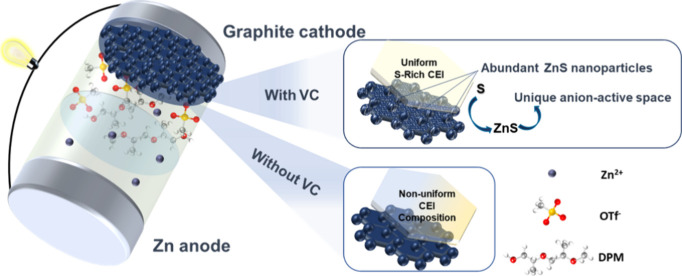

## Introduction

Renewable and clean energy sources, such as solar and wind energy, are urgently needed solutions to the scarcity of natural fossil resources and their environmental pollution. However, due to their intermittent nature, there is a corresponding need for high-performance electrochemical storage devices to store.^1^ Lithium-ion batteries, with high energy density, have been widely used in electronic devices and are considered promising power sources in energy storage systems. However, the safety concerns, cost, and availability of lithium resources increasingly hinder its large-scale application, prompting the search for alternative battery chemistries.^2^ Among these, dual-ion batteries have emerged as promising candidates due to their low material costs, high power capacity, and environmental friendliness, attracting increasing attention.^3^ Meanwhile, DIBs still face several challenges, such as limited energy density, structural failure, slow kinetics, and electrolyte decomposition.^4^

Extensive research efforts on DIBs have been undertaken in response to the challenges. Among these, the addition of appropriate electrolyte additives has proven to be an effective strategy. For instance, Cheng et al. reported on the novel use of trimethylsilyl phosphite (TMSP) as an electrolyte additive for DIBs.^3^ Their studies revealed that TMSP not only scavenges harmful byproducts generated during electrolysis but also facilitates the in situ formation of a thin, uniform CEI layer on graphite. As a result, electrochemical performance was significantly enhanced, with the batteries delivering a long lifespan of over 5000 cycles at 0.5 A g^–1^ and a high capacity of 90.1 mAh g^–1^ at 30 C rate. Similarly, Wang et al. demonstrated that lithium difluoro­(oxalato) borate (LiDFOB) as an additive helps form a robust ion-conductive CEI layer on the graphite cathode. This not only promotes the anion storage reaction but also suppresses side reactions and stabilizes the graphite structure, thereby achieving a high energy density of up to 422.7 Wh kg^–1^ at a current density of 100 mA g^–1^.^5^ These achievements underscore that the strategy of in situ formation of high-quality CEI through electrolyte additives can significantly enhance interface stability, reduce electrolyte decomposition, and improve ion transport dynamics, thus boosting battery performance. Despite the significant successes of electrolyte additives in improving the electrochemical performance of DIBs, further exploration of additive applications from other battery systems remains critical. This will help further uncover their potential applications in DIBs. Inspired by Yuan et al.’s research, which used ethyl vinyl sulfone (EVS) and fluoroethylene carbonate (FEC) as lithium metal battery electrolyte additives to reduce excessive decomposition of EVS and favor the formation of useful −SO_2_– components, as well as Yun et al.’s research, where silver hexafluorophosphate (AgPF_6_) was used to form uniformly distributed silver nanoparticles on the graphite cathode, creating more embedding sites and effectively improving Li^+^ insertion/extraction kinetics,^6,7^ which prompted us to further consider the role of electrolyte additives in DIBs. Specifically, we explore whether the additives in DIBs can suppress excessive decomposition while favoring the generation of useful components to optimize the CEI layer structure and whether their addition can facilitate the in situ formation of inorganic active nanosubstances on the graphite cathode surface with enhanced anion intercalation or storage site capacity. These explorations will deepen our understanding of the multifunction of additives in electrolytes and are crucial for enhancing the performance and stability of DIBs.

Herein, by incorporating a moderate amount (5 wt %) of vinylene carbonate (VC) into a mixed electrolyte of dipropylene glycol methyl ether (DPM) and water, a cathode electrolyte interface rich in sulfur components was successfully designed on the graphite cathode surface. This configuration significantly enhanced interface stability, suppressed electrolyte decomposition, and improved ion transport dynamics, while also providing protection to the graphite cathode structure. More importantly, the additive facilitated the formation of nanosized sulfide particles on the graphite cathode surface, which provided active sites for anion storage. X-ray photoelectron spectroscopy (XPS) demonstrated that the addition of VC effectively suppressed side reactions on the graphite surface and the decomposition of the electrolyte, while also confirming that the inclusion of VC promoted the formation of reduced state S^2–^ as nanoparticles and was conducive to forming a CEI rich in −SO_2_– components. These findings were further supported by time-of-flight secondary ion mass spectrometry (TOF-SIMS) tests. Additionally, high-resolution transmission electron microscopy (HRTEM) further confirmed the formation of nanosized sulfide particles on the graphite cathode surface. Scanning electron microscopy (SEM) and Raman spectroscopy showed that the additive effectively mitigated harmful structural damage to the graphite cathode. VC exhibited excellent performance in graphite-Zn battery.

## Results and Discussion

The preparation of the mixed electrolyte containing VC involves adding VC in a series of mass percentages (1, 5, 10 wt %) to a mixture of DPM and water (DPMW-1/5/10 VC; all abbreviations are shown in Table S1), followed by the addition of 4 M Zn­(OTf)_2_. The impact of different water-to-DPM ratios on battery performance was discussed in Figure S1. The experiment process is illustrated in Figure 1a. Figure 1b demonstrates the unique effect of the VC electrolyte additive on the graphite cathode. First, it can be observed that the addition of the additive effectively suppresses damage to the harmful structures of the graphite cathode. Further evidence can be obtained from the corresponding SEM images of the graphite cathode. As shown in Figure S2, the GP-50 (graphite cathode after 50 cycles) in DPMW-5VC maintains structural integrity consistent with the original graphite (GP), showing no signs of cracks, corrosion, or collapse. In contrast, the surface of GP-50 in DPMW exhibits signs of cracking damage. Second, the incorporation of the VC electrolyte additive has optimized the composition of the CEI, resulting in a more uniform distribution of elements and an increased content of S within the CEI components. This is substantiated by the EDS elemental mapping images of GP-50 in DPMW-5VC compared to GP-50 in DPMW. As illustrated in Figure 1c,d, the surface of GP-50 in DPMW-5VC shows a significantly higher content of S element compared to GP-50 in DPMW, with a more uniform distribution of O, F, S, and Zn on its surface. Additionally, the corresponding elemental atomic ratio charts and tables (Figure S3) further illustrate that GP-50 in DPMW-5VC has a higher S content on its surface. More importantly, compared to the GP surface and the graphite surface without electrolyte additive, the graphite flakes with 5 wt % VC added exhibit many fine particles within the lattice (as shown in the HRTEM image in Figure 2, and illustrated schematically in Figure 1b). These nanoscale active substances provide active storage space for anions. This is the primary reason for the stable increase in the discharge-specific capacity during the cycling process and for surpassing the discharge-specific capacity of the battery without the VC additive.

**Figure 1:**
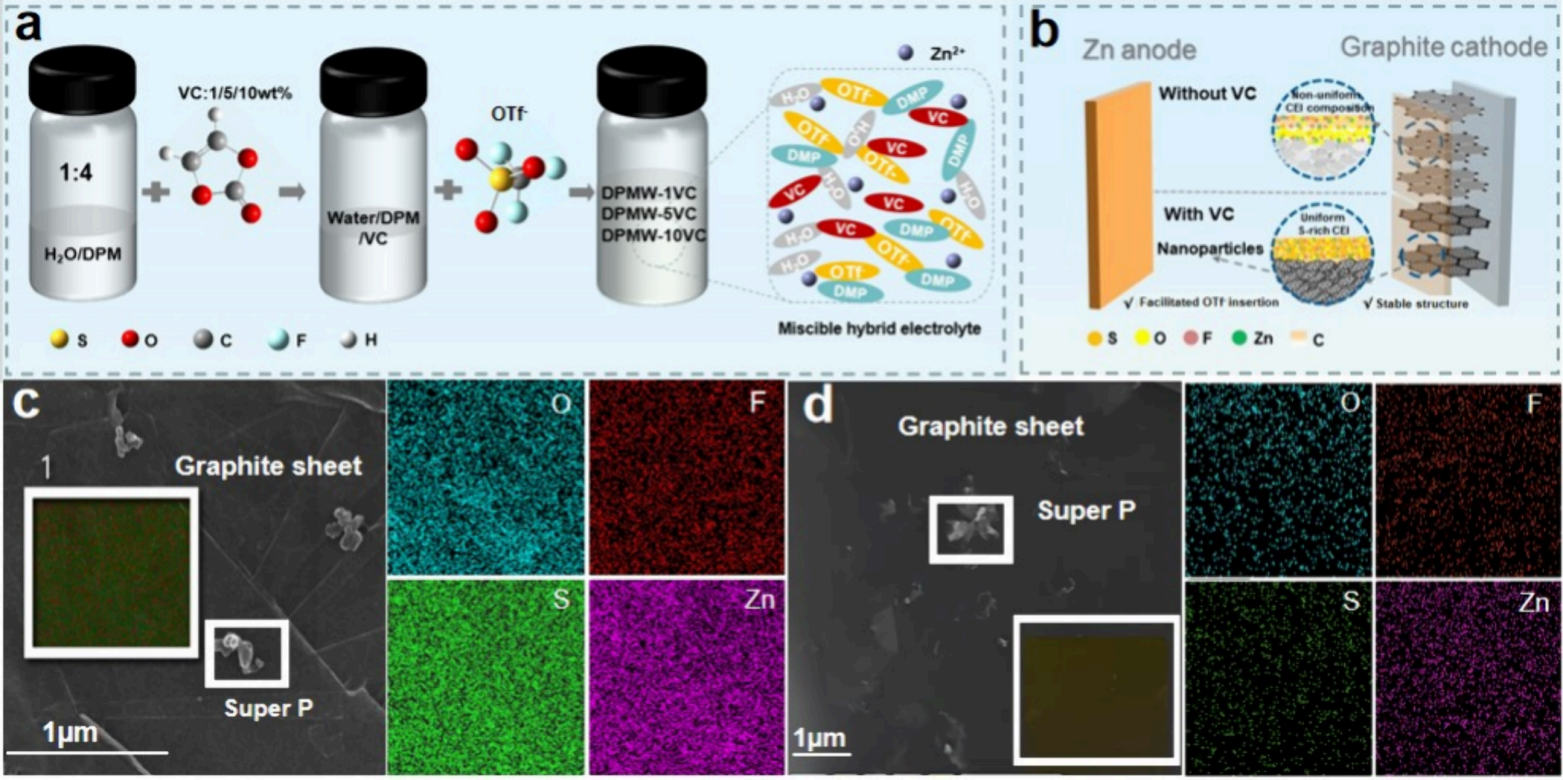
(a) Schemes of the electrolyte preparation process, and (b) unique functions of the electrolyte additive VC; (c) Energy dispersive spectroscopy (EDS) mapping images of GP-50 in DPMW-5VC; (d) EDS mapping images of GP-50 in DPMW.

**Figure 2:**
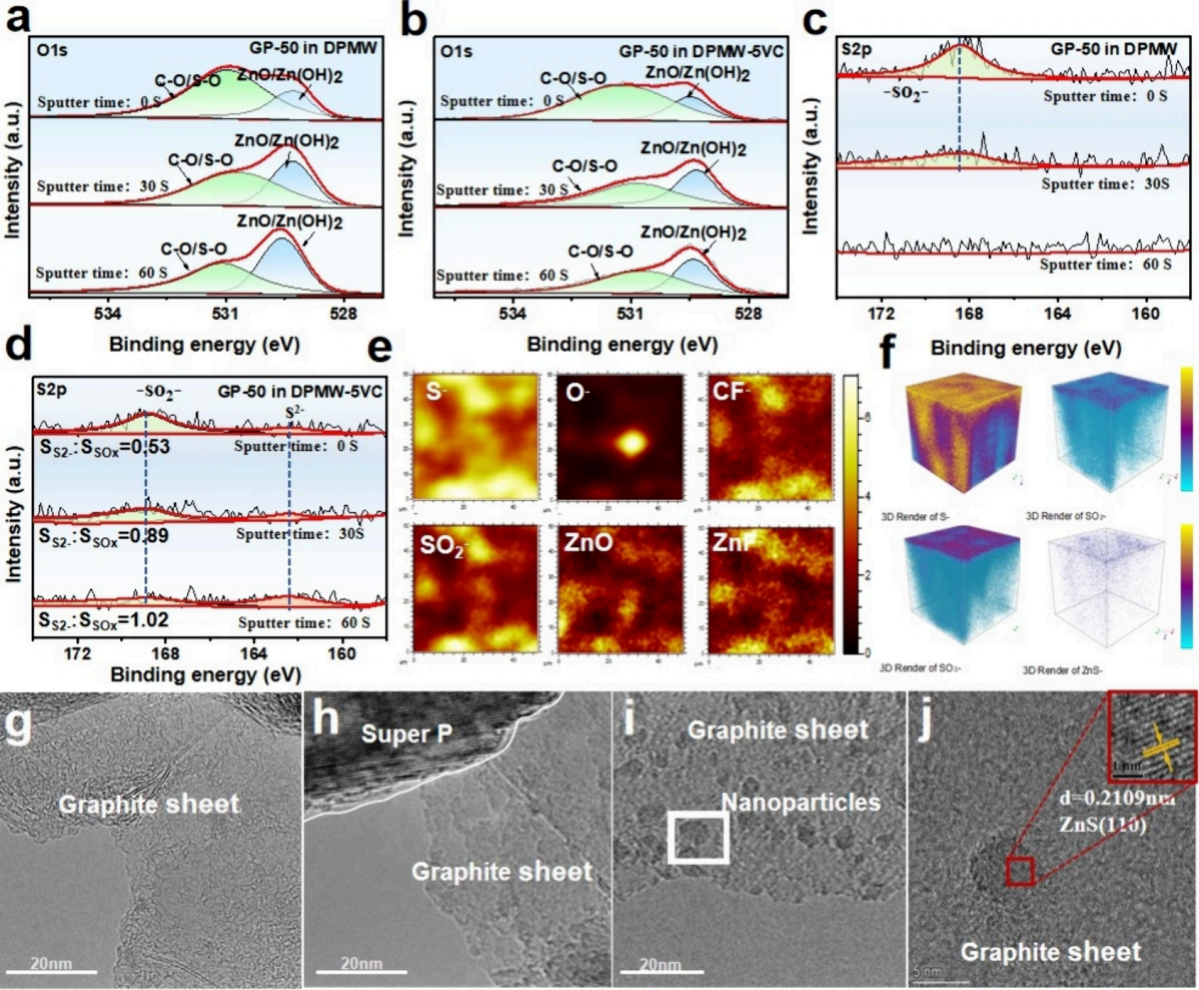
(a) O 1s XPS peak spectra of GP-50 in DPMW; (b) O 1s XPS peak spectra of GP-50 in DPMW-5VC; (c) S 2p XPS peak spectra of GP-50 in DPMW; (d) S 2p XPS peak spectra of GP-50 in DPMW-5VC; (e) 2D mapping spectra of TOF-SIMS; (f) Depth profile of secondary ion fragments on the surface of the graphite cathode; (g) HRTEM image of GP; (h) HRTEM image of GP-50 in DPMW; (i, j) HRTEM images of GP-50 in DPMW-5VC.

To gain a comprehensive understanding of the chemical environment evolution of CEI, XPS was performed on the surface of the graphite cathode in DPMW-5VC and DPMW mixed electrolytes at different cycle numbers. As shown in Figure S4, the XPS full spectra for GP-2 (graphite cathode after 2 cycles) in DPMW/DPMW-5VC clearly reveal signals for C, F, O, S, and Zn on the graphite surface. In the C 1s spectrum (Figure S5), the surface of GP-2 in DPMW detected C–C (284.31 eV), C–O (285.51 eV), and C=O (288.39 eV).^8–10^ These peaks correspond to the decomposition products of the electrolyte. Similarly, these signals were also detected on the surface of GP-50 in DPMW-5VC. However, a comparison revealed that the relative content of oxygen-containing organic groups in GP-50 in DPMW significantly increased, attributed to further decomposition of the electrolyte and oxidation of the graphite surface. In contrast, GP-50 in DPMW-5VC showed a much smaller XPS peak area for oxygen-containing organic groups, indicating that the decomposition of the electrolyte and the oxidation of the graphite surface were effectively suppressed, which corresponds to the results of the O 1s spectrum (Figure 2a,b).^11^ Additionally, compared to GP-50 in DPMW, GP-50 in DPMW-5VC exhibited a relatively lower intensity in the C 1s spectrum, further indicating a reduction in electrolyte decomposition.^6^ In the S 2p spectrum (Figure 2c,d), the amount of sulfur species bonded to oxygen (−SO_2_−) detected in GP-50 in DPMW-5VC decreases with increasing sputtering time, while the content of sulfur in the form of S^2–^ increases. In addition, by calculating the area ratio of S^2–^ to SO_*x*_, it is observed that this ratio gradually increases from 0.53 to 0.89, and eventually reaches 1.02 after 60 s of etching. This indicates that as the etching depth of the graphite surface increases, the relative ratio of S^2–^ to SO_*x*_ gradually increases, indicates that the addition of VC effectively promotes the formation of reduced sulfur species.

Furthermore, combined with the trends observed in the Zn 2p spectrum in Figure S6, it can be inferred that this transformation provides the necessary chemical conditions for the in situ formation of ZnS nanoparticles on the graphite surface. In contrast, in GP-50 in DPMW, the −SO_2_– peak gradually weakened and disappeared with increasing sputtering time, and no peak for S^2–^ was observed, indicating that in the absence of VC, sulfur on the graphite surface predominantly exists in an oxidized state, primarily concentrated in the surface layer. The presence of −SO_2_– in the inner layers of graphite is expected to lower the interfacial resistance, which is consistent with the observed lower Rct for GP-50 in DPMW-5VC.^6^

In the F 1s spectrum (Figure S6), the peaks for C–F and ZnF_2_ were located at 688.14 and 684.17 eV, respectively, attributed to the decomposition products of Zn­(OTf)_2_.^10^ Compared to GP-50 in DPMW, GP-50 in DPMW-5VC exhibited lower intensities, indicating that the addition of VC not only facilitated the decomposition of OSCF_3_ from the main salt to form in situ sulfur-containing species but also effectively suppressed the decomposition of CF_3_ groups.

Furthermore, the chemical composition of the CEI layer at different depths was analyzed by conducting depth profiling on the graphite surface. The results showed that the CEI of GP-50 in DPMW-5VC had a more uniform vertical distribution (from surface to interior). This uniformity can be demonstrated by the atomic concentration percentage maps of different elements from the surface to the interior of the graphite cathode. As shown in Figure S7, the content of C increased with increasing sputtering time (possibly due to DPM adsorption on the graphite surface, forming a carbon-containing layer), while the content of F decreased. A slight decrease in O and S was also observed, but these changes were consistent with the graphite surface, indicating the uniformity of the CEI on the graphite cathode. The XPS data from the second, 50th, and 100th cycles are also combined to compare the evolution of the CEI components, as discussed in Figure S8. The XPS results confirm that the VC additive is crucial for suppressing electrolyte decomposition, promoting the formation of ZnS nanoparticles on the graphite surface, achieving vertical uniform distribution of the CEI layer, and improving the interfacial characteristics between the electrode and electrolyte.

Further analysis of the chemical element distribution and compositional changes of the CEI on the cycled graphite cathode materials was conducted using TOF-SIMS. However, due to the uneven and irregular micronanoparticles that comprise graphite, highly ordered pyrolytic graphite (HOPG) with a smooth and intact surface was chosen as a substitute. The 2D mapping spectra from TOF-SIMS (Figure 2e) indicate that more nonoxygen-bound S^–^ was detected in HOPG in DPMW-5VC, creating necessary conditions for the in situ formation of ZnS nanoparticles, consistent with the previous XPS analysis results. The CF^–^ and ZnF^–^ components primarily originate from the decomposition of Zn­(OTf)_2_, and these components are evidently less present on the surface of GP in DPMW-5VC, while the S^–^ content is higher. This observation further confirms that the addition of VC facilitates the decomposition of OSCF_3_ from the main salt to form in situ sulfur-containing species, while effectively suppressing the decomposition of CF_3_ groups. Additionally, the detected O^–^ and ZnO components on the graphite surface were minimal, further validating the suppression of side reactions, particularly the oxidation of the graphite surface.

The 3D rendering and TOF-SIMS chemical imaging provided enhanced visualization of the concentration gradient of materials in the CEI layer after cycling.^12^ Figure 2f shows the depth profile of secondary ion fragments on the surface of the HOPG material. The results indicate that more nonoxygen-bound S^–^ was detected in both the inner and outer layers of the CEI in HOPG in DPMW-5VC, and abundant −SO_*n*_– fragments were also found in the inner layer. The presence of the sulfur layer may enhance the stability of the CEI by inhibiting the continuous decomposition of the electrolyte on the electrode surface, which is consistent with the previous XPS results. The 3D renderings of other elements, such as O and F, are also shown in Figure S9.

HRTEM has demonstrated that the addition of the electrolyte additive VC can induce the formation of nanoparticles on the graphite surface. As shown in Figure 2g–i, a comparison with GP reveals that numerous fine particles are present within the lattice of GP-50 in DPMW-5VC graphite sheets. To further confirm the composition of these nanoparticles, the lattice spacings of the nanoparticles were measured using the fast Fourier transform (FFT). As shown in Figures 2j and S10, the lattice spacings of these nanoparticles are 0.237 and 0.2109 nm, corresponding to the (101) plane of ZnO and the (110) plane of ZnS, respectively, with ZnS being more abundant.^13,14^ Therefore, it can be concluded that the nanoparticles on the graphite surface are primarily ZnS nanoparticles.

Detailed electrochemical characterizations were conducted using cyclic voltammetry (CV) to further determine the origin of the ZnS nanoparticles. Initially, CV tests explored whether the nanoscale sulfides on the graphite surface were provided by the anion OTf^–^ or Zn^2+^. Figure S11a shows the CV curve of the battery from the open circuit voltage to 2.4 V and then from 2.4 to 0.5 V in the first cycle. The oxidation peak at 1.53 V can be attributed to the intercalation of anions and solvent, suggesting that the nanoparticles on the graphite surface could be attributed to the decomposition of the solvent within the graphite and the formation with partially deintercalated anions. The reduction peak at 1.48 V could be attributed to partially deintercalated anions. Figure S11b shows the CV curve for the first cycle of the battery, measured from the open-circuit voltage to 0.5 V and then from 0.5 to 2.4 V. The comparison further demonstrates that the oxidation peak at 1.53 V is not affected by the reduction process, indicating that the nanoscale sulfide active material on the graphite surface is formed through the decomposition of the solvent in conjunction with the anions, with minimal direct involvement of Zn^2+^. Moreover, the CV curves in Figure S12 display the altered charge transfer kinetics due to the presence of ZnS nanoparticles. Specifically, there were noticeable shifts in the redox peaks before and after cycling and changes in peak height and width, which may be attributed to a change in the intercalated anions (previously OTf^–^, possibly OTf^–^ and S^2–^ after cycling), affecting the potential for charge transfer and enhancing the charge storage capacity.

Raman spectroscopy was utilized to analyze the crystallinity of graphite and further investigate the protective effects of VC on the graphite structure. The *I*_D_/*I*_G_ peak ratio is commonly used to evaluate the degree of graphitization in carbon materials, where a higher *I*_D_/*I*_G_ ratio indicates more severe damage to the graphite.^15–17^ As shown in Figure S13, the *I*_D_/*I*_G_ ratio of GP-50 in DPMW shows little change compared to GP, indicating that the ion intercalation and deintercalation efficiency of the graphite electrode is relatively low and thus has a limited impact on the graphite structure. In contrast, the *I*_D_/*I*_G_ ratio of GP-50 in DPMW-5VC (*I*_D_/*I*_G_ = 0.238) is significantly reduced, which could be due to the strong interaction between VC and the graphite surface, promoting the graphitization reconstruction of the graphite surface. The improved graphitization not only enhances its conductivity but also facilitates ion intercalation and deintercalation, further optimizing the electrochemical performance of the battery. This result further confirms that the VC additive not only optimizes the chemical composition and electrochemical reactions of the CEI but also protects the physical structure of the electrode, significantly improving its conductivity and structural stability.

After discussing the multiple positive effects of the VC additive, a full cell based on a graphite cathode and Zn anode was fabricated to assess the feasibility of the 4 M DPMW-5VC electrolyte. The working mechanism, as shown in Figure 3a, involves the migration of Zn^2+^ ions from the electrolyte to the Zn anode during charging, while OTf^–^ anions from the electrolyte intercalate into the graphite cathode. During discharging, Zn^2+^ and OTf^–^ ions respectively release back into the electrolyte from the Zn anode and graphite cathode. At the same time, the nonreturning anions and cointercalated solvent molecules decompose at the graphite cathode and combine with Zn^2+^ to form ZnS.

**Figure 3:**
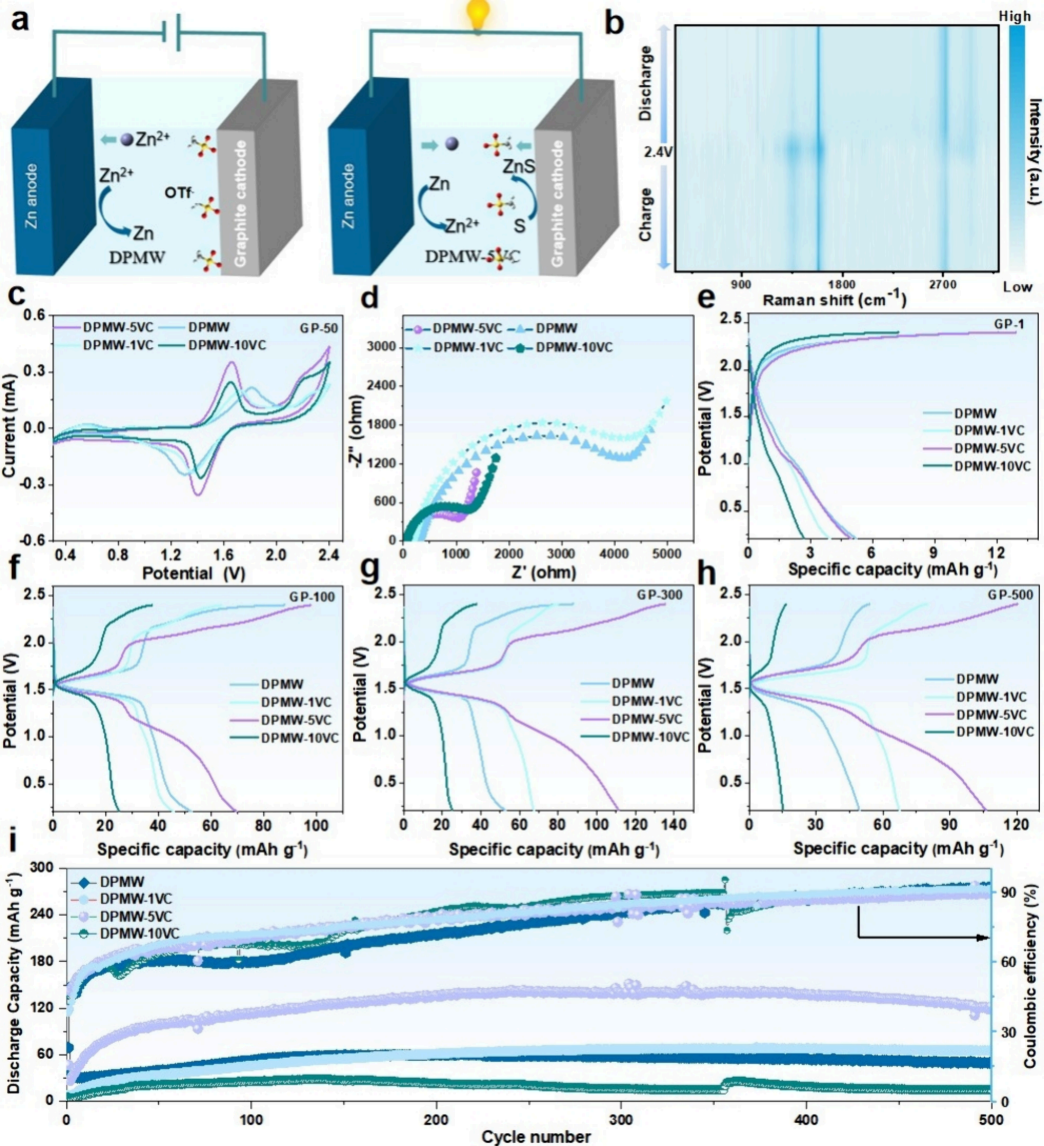
(a) Working principle of graphite-Zn battery; (b) In situ Raman spectra of the charging and discharging process of GP-50 in DPMW-5VC; (c) CV curves of GP-50 in DPMW and GP-50 in DPMW-1/5/10VC, with a scan rate of 0.8 mV s^–1^; (d) Nyquist plots of GP-50 in DPMW and GP-50 in DPMW-1/5/10VC; (e–h) Charging/discharging curves of GP in DPMW and GP in DPMW-1/5/10VC at different cycling counts (first, 100th, 300th, 500th cycles); (i) Comparison of cycling performance of GP-50 in DPMW and GP-50 in DPMW-1/5/10VC.

To better understand the working mechanism of the graphite-Zn battery, in situ Raman and ex-situ XPS characterizations were further employed. Figure 3b shows the in situ Raman spectrum of the graphite cathode during the 50th cycle of the graphite-Zn battery. It can be observed that the graphite G band peak at 1580 cm^–1^ gradually broadens during charging and recovers after complete discharge, corresponding to the intercalation and deintercalation process of OTf^–^ anions.^18,19^ In the XPS tests, as displayed in Figure S14a, the Zn 2p spectrum shows two strong Zn^2+^ peaks at 1021.78 and 1044.88 eV when the battery is fully discharged to 0.2 V, and the intensity of these peaks decreases when fully charged to 2.4 V, indicating the electrochemical intercalation and deintercalation of Zn.^20–22^ Concurrently, in the S 2p spectrum (Figure S14b), an S^2–^ signal is detected at 164.2 eV when the battery is fully discharged to 0.2 V, and the signal for S weakens upon charging to 2.4 V, indicating the transformation of S and ZnS during the charging and discharging process.^23^

After understanding these microscopic mechanisms, the effect of different VC concentrations on battery performance was further evaluated through CV tests. Figures 3c and S15 display the CV curves for GP-2/50 in DPMW and GP-2/50 in DPMW-1/5/10VC at a scan rate of 0.8 mV s^–1^, with a voltage range of 0.2–2.4 V. The four electrolytes (DPMW, DPMW-1/5/10VC) exhibited similar CV curves, indicating comparable redox behavior. After 2 cycles, the main oxidation and reduction peaks for the three electrolytes (DPMW, DPMW-1VC, DPMW-5VC) were located at 1.63, 1.58, 1.61 V and 1.44, 1.45, 1.43 V, respectively. No significant oxidation or reduction peaks were observed in the DPMW-10VC electrolyte, indicating that the embedding and de-embedding processes of the anions are restricted. After undergoing 50 cycles, the main oxidation and reduction peaks for all four electrolytes (DPMW, DPMW-1/5/10VC) were found at 1.8, 1.73, 1.65, 1.65 V and 1.3, 1.35, 1.40, 1.42 V, respectively. The differences between the main oxidation and reduction peaks were 0.5, 0.38, 0.25, and 0.23 V, respectively. The results indicate that the oxidation and reduction peak positions in the DPMW-5VC and DPMW-10VC electrolytes nearly overlap, suggesting that the charge and discharge processes of the battery in DPMW-5VC and DPMW-10VC electrolytes are reversible. In contrast, the batteries with DPMW and DPMW-1VC electrolytes exhibited irreversible oxidation peaks at 0.57, 0.9 V, 0.55 and 0.897 V, respectively. This may lead to reduced cycling stability and accelerated electrode degradation, ultimately shortening the battery’s lifespan. Moreover, compared to the other three electrolytes, the CV curves of the battery using DPMW-5VC showed larger integral areas and nearly unchanged potentials of the oxidation and reduction peaks. Therefore, the graphite cathode in the DPMW-5VC electrolyte demonstrated better electrochemical activity and enhanced energy storage performance after 50 charge–discharge cycles.^24–26^ It can be seen from the middle-frequency region in Figure 3d that the ionic impedance of the DPMW-5VC electrolyte (1184 Ω) is much lower than that of the graphite electrode in the DPMW electrolyte (4853 Ω), suggesting that the addition of VC significantly reduces the whole battery resistance, resulting in higher ionic conductivity (1.88929 × 10^–5^ S cm^–1^, compared to 0.46132 × 10^–5^ S cm^–1^ of DPMW electrolyte) (Figure S16 and Table S2).

Additionally, we measured the long-term stability of the battery after cycling at a current density of 100 mA g^–1^ following a precycle at a current density of 50 mA g^–1^ (to form the CEI). Figure 3e–h illustrate the variations in the GCD curves of the graphite-Zn battery in different electrolytes (DPMW, DPMW-1/5/10VC) at different cycles (first, 100th, 300th and 500th cycles). For batteries with different amounts of electrolyte additive, characteristic charge–discharge plateaus corresponding to the intercalation/deintercalation of OTf^–^ from graphite cathode can be observed. During the cycling, the battery in the DPMW-5VC electrolyte exhibited relatively small voltage drops after the first cycle and throughout multiple cycles, with a stable voltage curve, indicating a lower voltage drop. In contrast, batteries in DPMW, DPMW-1VC, and DPMW-10VC electrolytes experienced rapid voltage drops after both the first cycle and multiple cycles, demonstrating a relatively larger voltage drop. This indicates that the addition of VC in the electrolyte can achieve more effective charging at the first cycle. We also compared their ionic diffusion coefficients by using galvanostatic intermittent titration technique (GITT) during the charge and discharge (see Figure S17). The DPMW-5VC exhibits a slightly higher log D value, where D represents the ionic diffusion coefficient.

Figure 3i shows the graphite-Zn battery’s cycling stability and CE comparison with different VC contents. The battery in the DPMW electrolyte underwent 500 cycles, with the discharge-specific capacity dropping to 49.44 mAh g^–1^. However, the introduction of VC additives improved the cycling performance of the graphite cathode. Although the capacity of the battery using the DPMW-1VC electrolyte remained stable, its discharge-specific capacity was still relatively lower compared to the battery using the DPMW-5VC electrolyte, which maintained a corresponding discharge-specific capacity of 118 mAh g^–1^ after 500 cycles, with a CE of 89.42%. However, when the VC content was further increased to 10%, the capacity retention and average CE of the battery even dropped below those without VC. This could be due to the fact that the high concentration of electrolyte additives caused more solvent molecules to cointercalate, occupying sites that could have been available for the OTf^–^ anion. As a result, during the initial cycles, OTf^–^ anions accumulated on the surface or in the interlayers of the graphite cathode, preventing effective intercalation into the graphite layers. Therefore, in the CV curves after two cycles (Figure S15), the high-concentration electrolyte additives did not show distinct oxidation and reduction peaks for DPMW-10VC. This phenomenon also affected the effective intercalation of OTf^–^ in subsequent cycles, which in turn impacted the capacity recovery and led to capacity fading during cycling. On the other hand, the addition of VC promoted the decomposition of the main salt (as evidenced by the XPS and TOF-SIMS data in Figure 2). However, excessive VC may lead to the overdecomposition of the main salt, resulting in the formation of a thick CEI layer on the cathode surface, which further affects the battery performance, reaffirming the optimized advantages of adding 5 wt % of the VC electrolyte additive. The rate performance of the battery using the electrolyte with 5 wt % VC is presented in Figure S18. The effects of DPMW-5VC on the Zn anode were further investigated using SEM and symmetric cell testing, as shown in Figure S19. Finally, we also conducted a comparative analysis of performance data from other reported work on electrolyte additives in the literature (Table S3), further demonstrating the advantages of VC electrolyte additives in enhancing battery performance.

Through the aforementioned experimental analysis, it has been shown that the electrolyte additive VC can improve battery performance. To explore the mechanism behind these results, density functional theory (DFT) calculations were performed by using Gaussian 09.^27^ As shown in Figures 4a and S20a, the values and images of the lowest unoccupied molecular orbital (LUMO) and the highest occupied molecular orbital (HOMO) of the electrolyte components are illustrated. Figure 4a simulates the redox trends of Zn­(OTf)_2_, VC, DPM, and H_2_O. The HOMO energy level of VC (−7.2 eV) is slightly higher than that of DPM (−7.22 eV) and H_2_O (−8.88 eV), indicating that VC will be preferentially oxidized on the surface of the cathode, effectively preventing the decomposition of DPM and H_2_O solvents, and avoiding further side reactions in subsequent cycling processes through protective product layer.

**Figure 4:**
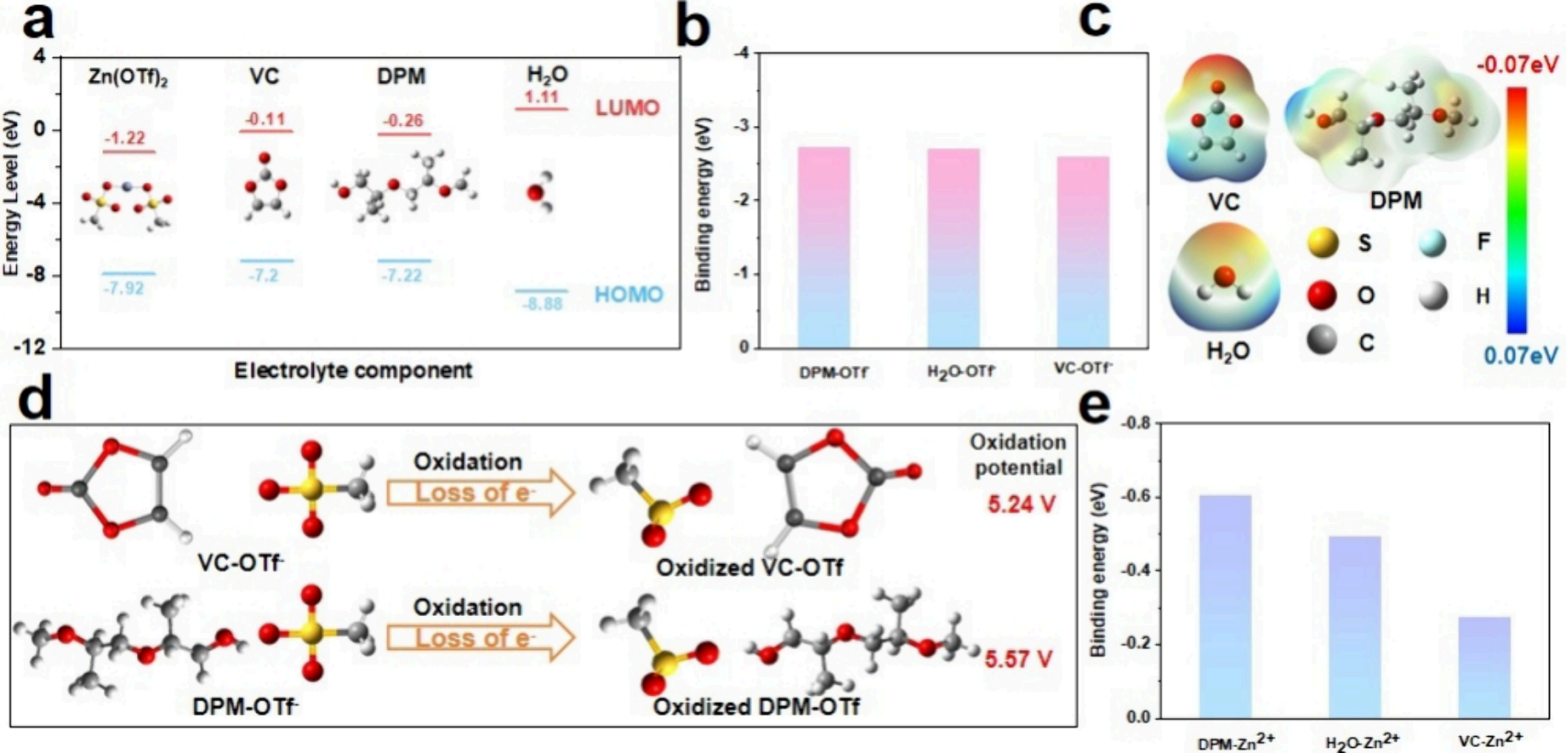
(a) DFT-calculated HOMO and LUMO values for Zn­(OTf)_2_, VC, DPM, and H_2_O; (b) Binding energies of DPM-OTf^–^, H_2_O-OTf^–^, and VC-OTf^–^ clusters; (c) ESP density distribution for electrolyte additives and solvents; (d) Calculated oxidation potentials for VC-OTf^–^ and DPM-OTf^–^ clusters; (e) Binding energies of DPM-Zn^2+^, H_2_O–Zn^2+^, and VC-Zn^2+^ clusters.

Considering that anions may participate in the oxidation process of liquid electrolytes, it is necessary to consider the clusters formed by OTf^–^ and other electrolyte components when discussing the oxidation sequence.^28,29^ As shown in Figure 4c, the binding sites determined by the electrostatic potential (ESP) are displayed. Therefore, based on ESP, the binding energies (Table S5) and oxidation potentials (Table S6) of the solvent/additive-OTf^–^ clusters were calculated using DFT. The binding energies between VC, DPM, H_2_O, and the OTf^–^ anion indicate that there is a strong association ability between VC, DPM, H_2_O, and the ion (Figure 4b), suggesting that the OTf^–^ anion may participate in their oxidation process. Subsequently, the oxidation potentials of the VC-OTf^–^, DPM-OTf^–^, and H_2_O-OTf^–^ clusters were calculated (Figure 4d). The results show that the oxidation potential of the VC-OTf^–^ cluster (5.24 V) is lower than that of the DPM-OTf^–^ cluster (5.57 V) and the H_2_O-OTf^–^ cluster (5.90 V) (Figure S20). It also indicates that the VC additive will be preferentially oxidized on the graphite positive electrode side and form a protective CEI film, selectively promoting the decomposition of the OSCF_3_ group to form sulfur-containing substances. Meanwhile, the CEI film formed by VC on the electrode surface can effectively inhibit the decomposition of active groups like CF_3_.

Additionally, considering that the additive may participate in the solvation structure of Zn^2+^, the binding energies between Zn^2+^ and the electrolyte components were further calculated (Figure 4e).^30^ The results show that the binding energy between VC and Zn^2+^ is lower than that between the solvent and Zn^2+^, indicating that VC participates in the solvation structure of Zn^2+^, creating the preconditions for the in situ formation of ZnS particles on the cathode lattice.

## Conclusions

In summary, by incorporating the environmentally friendly and cost-effective VC additive into a 4 M DPM/water mixed electrolyte system, we have achieved long-term stable cycling in graphite-Zn DIBs. Specifically, the addition of VC facilitated the formation of a sulfur-rich layer on the graphite cathode surface, optimizing the composition of the CEI. This significantly enhanced interface stability, inhibited electrolyte decomposition, and improved ion transport dynamics, while also providing protection to the graphite cathode structure. More importantly, the introduction of the electrolyte additive promoted the formation of nanosized sulfide particles on the graphite cathode surface. These nanosized sulfide particles provided active sites for anion storage, thus ensuring the excellent electrochemical performance of the DIBs. Overall, this work demonstrates the significant potential of electrolyte additives in enhancing battery performance. It provides new insights into how these additives can optimize the composition of the graphite cathode CEI and promote the formation of inorganic nanosized particle hosts, thereby improving the performance of DIBs. This advances the practical application of DIBs in future sustainable energy storage systems.

## Experimental Section

### Electrode Preparation and Battery Assembly

The method for preparing the cathode slurry involves using expanded graphite powder (EG, 10–30 μm, 99.9% carbon) as the active material, conductive carbon black (Super P) as the conductive agent, and polytetrafluoroethylene emulsion (PTFE) as the binder. These components are mixed in a mass ratio of 8:1:1 in 1-methyl-2-pyrrolidone (NMP) and stirred for 12 h on a magnetic stirrer to obtain a homogeneous slurry. The resulting slurry is then spread onto a stainless-steel mesh using a scraper. The electrodes are dried in a vacuum oven at 60 °C for 10 h, cut into discs with a diameter of 16 mm, and then roll-pressed. The average mass of the electrodes is about 2 mg. The anode is 99.995% high-purity metallic zinc foil, which is also cut into 16 mm discs for assembling the battery. These electrodes are assembled into CR2032 coin cells, using a glass microfiber separator with a diameter of 19 mm and a thickness of 4.5 mm. Approximately 150 μL of electrolyte is used to wet the separator. Finally, the battery is sealed using a hydraulic crimping machine at a pressure of 500 psi.

### Electrochemical Tests

Constant current charge–discharge tests are conducted using Neware battery testers within a voltage window from 0.2 to 2.4 V. CV measurements are performed using a CHI660E electrochemical workstation at a scan rate of 0.8 mV s^–1^. Electrochemical impedance spectroscopy (EIS) is conducted on batteries at their initial state and after various cycle numbers using the CHI660E electrochemical workstation, with an AC potential amplitude of 5 mV and a frequency range from 100 kHz to 0.01 Hz.

### Electrochemical Characterization

X-ray photoelectron spectroscopy (XPS) and time-of-flight secondary ion mass spectrometry (ION-TOF TOF.SIMS5) are used to investigate the composition of the CEI on and within the graphite surface, as well as the reaction mechanisms of the battery. The structure of the graphite cathode after cycling is analyzed using a Zeiss JEM-6510 field emission scanning electron microscope (FE-SEM) and InVia laser confocal Raman spectroscopy (from Renishaw). Transmission electron microscopy (TEM) images of the graphite electrodes after cycling, both fully charged and discharged, are collected using the Thermo Fisher Talos F200X G2.

### Calculation Method

The highest occupied molecular orbital and lowest unoccupied molecular orbital of DPM, VC, H_2_O, and Zn­(OTf)_2_ were calculated using the Gaussian 09 program.^31^ All optimized geometries and frequency calculations are performed using the B3LYP function and the 6-311+G­(d,p) standard basis. The continuous solvent model (SMD) was included in the calculations, in which the dielectric constant of the mixed electrolyte was calculated to be 33.72 based on the volume ratio (Table S4) to obtain the electrostatic potential (ESP) mapping by further calculating the Gaussian check file. The binding energy can be calculated using the following eq 1:^30^

E(bindingenergy)=E(solvent/additivewithion)−E(solvent/additive)−E(ion) 1

In addition, the oxidation potentials of VC, DPM, and H_2_O are determined using eq 2 as follows:^30^

$$\mathrm{EOX}({\mathrm{Zn}}^{2+}/\mathrm{Zn})=[G(M^{+})-G(M)]/F-1.4\;V$$ 2

where *G*(M) represents the free energy of the electrolyte component M; *G*(M^+^) represents the oxidation of M at 298.15 K, and *F* represents the Faraday constant.

## Supplementary Material



## Data Availability

The data that support the findings of this study are openly available in zenodo.org at 10.5281/zenodo.14886235.

## Notes

Nomenclature table of electrolyte systems, battery performance plots with different H_2_O/DPM ratios, SEM images of GP and GP-50 in DPMW/DPMW-5VC electrolytes, EDS spectra of GP-50 in DPMW/DPMW-5VC electrolytes, XPS survey spectra of GP-2 in DPMW/DPMW-5VC electrolytes, XPS analysis of C 1s orbitals for GP-2/50 in DPMW/DPMW-5VC electrolytes, XPS analysis of Zn 2p and F 1s orbitals for GP-50 in DPMW/DPMW-5VC electrolytes, XPS depth profiling of GP-50 in DPMW/DPMW-5VC electrolytes, XPS analysis of C 1s, F 1s, O 1s, Zn 2p, and S 2p orbitals for GP-2/50/100 in DPMW/DPMW-5VC electrolytes, TOF-SIMS depth profile analysis of HOPG surface, HRTEM images of GP-50 in DPMW/DPMW-5VC electrolytes, EDX elemental mapping images of graphite cathode after cycling in DPMW-5VC electrolyte, CV curves of graphite-Zn batteries in DPMW-5VC electrolyte during initial cycles (forward/reverse) and after 2/50 cycles, Raman spectra of GP and GP-50 in DPMW/DPMW-5VC, XPS analysis of Zn 2p and S 2p orbitals for graphite-Zn batteries in DPMW-5VC electrolyte at different charge/discharge states, CV curves of graphite-Zn batteries after 2 cycles in DPMW/DPMW-1VC/DPMW-5VC/DPMW-10VC electrolytes, ionic conductivity plots of graphite-Zn batteries in DPMW/DPMW-1VC/DPMW-5VC/DPMW-10VC electrolytes, GITT curves of batteries with DPMW/DPMW-5VC/DPMW-10VC electrolytes during charge/discharge processes, detailed ionic conductivity calculations, rate capability plots of graphite-Zn batteries in DPMW-5VC electrolyte, SEM images of pristine Zn foil surface and Zn anode surfaces after cycling in DPMW/DPMW-5VC electrolytes, long-term stability tests of Zn//Zn symmetric cells in DPMW/DPMW-5VC electrolytes, LUMO and HOMO diagrams of electrolyte components, calculated oxidation potential diagrams of H_2_O-OTf^–^ clusters, performance comparison tables between this study and other studies, calculations of mixed solvent dielectric constants, calculations of binding energies and oxidation potentials for various clusters in electrolytes (PDF)

## References

[1] Guan S., Peng Q., Guo X., Zheng Y., Tang Y. (2024). A review of the advances
and prospects of aqueous dual-ion batteries. Chem. Eng. J..

[2] Wu M., Zhang G., Yang H., Liu X., Sun S. (2021). Aqueous Zn-based rechargeable batteries: Recent
progress and future
perspectives. InfoMat.

[3] Cheng Z., Guo L., Dong Q., Wang C., Qian Y. (2022). Highly
durable and ultrafast cycling of dual-ion batteries via in situ construction
of cathode-electrolyte interphase. Adv. Energy
Mater..

[4] Li J., Hui K. S., Hui K. N. (2022). Review of electrolyte
strategies for competitive dual-ion batteries. Mater. Today Sustainability.

[5] Wang Y., Zhang Y., Wang S. (2021). Ultrafast charging and
stable cycling dual-ion batteries enabled via an artificial cathode-electrolyte
interface. Adv. Funct. Mater..

[6] Yuan X., Dong T., Liu J., Zhang H. (2023). Bi-affinity
electrolyte optimizing high-voltage lithium-rich manganese oxide battery. Angew. Chem., Int. Ed..

[7] Yun J., Wang Y., Gao T., Zheng H., Zheng H. (2015). In-situ electrochemical
coating of Ag nanoparticles onto graphite
electrode with enhanced performance for Li-ion batteries. Electrochim. Acta.

[8] Di S., Nie X., Ma G., Zhang N. (2021). Zinc anode stabilized
by an organic-inorganic hybrid solid electrolyte interphase. Energy Storage Mater..

[9] Nguyen D., Eng A. Y. S., Ng M., Kumar V., Seh Z. W. (2020). A high-performance magnesium
triflate-based electrolyte for rechargeable
magnesium batteries. Cell Rep. Phys. Sci..

[10] Li D., Cao L., Deng T., Liu S., Wang C. (2021). Design of a solid electrolyte
interphase for aqueous Zn batteries. Angew.
Chem., Int. Ed..

[11] Wang Y., Zhang Y., Duan Q., Lee P., Yu D. Y. W. (2020). Engineering cathode-electrolyte interface
of graphite
to enable ultra long-cycle and high-power dual-ion batteries. J. Power Sources.

[12] Chen Y., Zhao W., Zhang Q., Yang G., Peng C. (2020). Armoring LiNi_1/3_Co_1/3_Mn_1/3_O_2_ ccathode with reliable
fluorinated organic-inorganic hybrid
interphase layer toward durable high rate battery. Adv. Funct. Mater..

[13] Flores G., Carrillo J., Luna J. A., Martínez R., Rabanal M. E. (2014). Synthesis, characterization and photocatalytic
properties of nanostructured ZnO particles obtained by low temperature
air-assisted-USP. Adv. Powder Technol..

[14] Cai R., Yang D., Zhang L., Qiu L., Tan W. (2016). A facile process for the preparation of three-dimensional
hollow
Zn­(OH)_2_ nanoflowers at room temperature. Chem.—Eur. J..

[15] Zhang K., Li D., Shao J., Jiang Y., Zheng H. (2023). Ultrafast
charge and long life of high-voltage cathodes for dual-ion batteries
via a bifunctional interphase nanolayer on graphite particles. Small.

[16] Fu Y., Cao C., Song W., Li B., Chen J. (2024). Self-assembly strategy
for constructing porous boron and nitrogen co-doped carbon as an efficient
ORR electrocatalyst toward zinc-air battery. Chem.—Eur. J..

[17] Xie X., Fan W., Zhang J., Ma R., Wang C. (2023). Regeneration
of graphite anode from spent lithium iron phosphate batteries: Microstructure
and morphology evolution at different thermal-repair temperature. Powder Technol..

[18] Zhang N., Dong Y., Wang Y., Wang Y., Cheng F. (2019). Ultrafast rechargeable
zinc battery based on high-voltage graphite
cathode and stable nonaqueous electrolyte. ACS
Appl. Mater. Interfaces.

[19] Huang Z., Hou Y., Wang T., Zhao Y., Zhi C. (2021). Manipulating anion
intercalation enables a high-voltage aqueous dual ion battery. Nat. Commun..

[20] Vijayakumar S., Lee S., Ryu K. (2015). Synthesis
of Zn_3_V_2_O_8_ nanoplatelets for lithium-ion
battery and supercapacitor applications. RSC
Adv..

[21] Chang X., Li K., Qiao X., Xiong Y., Xue Q. (2021). ZIF-8
derived ZnO polyhedrons decorated with biomass derived nitrogen-doped
porous carbon for enhanced acetone sensing. Sens. Actuators, B.

[22] Baynosa M. L., Mady A. H., Nguyen V. Q., Kumar D. R., Shim J. (2020). Eco-friendly synthesis
of recyclable mesoporous zinc ferrite@reduced
graphene oxide nanocomposite for efficient photocatalytic dye degradation
under solar radiation. J. Colloid Interface
Sci..

[23] Li W., Wang K., Jiang K. (2020). A low cost
aqueous Zn-S battery realizing
ultrahigh energy density. Adv. Sci..

[24] Gu J., Yuan Z., Wang H., Shen J., Hu Y. (2022). Local
protonation of polyaniline induced by nitrogen-doped carbon
skeleton towards ultra-stable Zn-organic batteries with a dual-ion
insertion/extraction mechanism. Chem. Eng. J..

[25] Li H., Kurihara T., Yang D., Watanabe M., Ishihara T. (2021). A novel aqueous
dual-ion battery using concentrated bisalt electrolyte. Energy Storage Mater..

[26] Chen M., Xia X., Qi M., Yuan J., Chen Q. (2015). Controllable
synthesis of hierarchical porous nickel oxide sheets arrays as anode
for high-performance lithium ion batteries. Electrochim. Acta.

[27] Frisch, M. J. ; Trucks, G. W. ; Schlegel, H. B. ; Scuseria, G. E. ; Robb, M. A. Gaussian 09, Revision D.01; Gaussian, Inc.: Wallingford, CT, 2013.

[28] Fan Z., Zhou X., Qiu J., Yang Z., Chou S. (2023). Sulfur-rich additive-induced
interphases enable highly stable 4.6
V LiNi_0.5_Co_0.2_Mn_0.3_O_2_||graphite
Pouch Cells. Angew. Chem., Int. Ed..

[29] Xing L., Wang C., Li W., Xu M., Zhao S. (2009). Theoretical insight into oxidative decomposition of
propylene carbonate
in the lithium ion battery. J. Phys. Chem. B.

[30] Li J., Fan Z., Ye H., Zheng J., Zeng R. (2024). Novel
sulfur-based electrolyte additive for constructing high-quality sulfur-containing
electrode-electrolyte interphase films in sodium-ion batteries. Chem. Eng. J..

[31] Yaqoob M., Gul S., Zubair N. F., Iqbal J., Iqbal M. A. (2020). Theoretical calculation
of selenium N-heterocyclic carbene compounds through DFT studies:
Synthesis, characterization and biological potential. J. Mol. Struct..

