# Supplementary material for: In Situ Modification of Sulfur-Based Cathode Electrolyte Interphases for Boosting Zinc/Graphite Dual-Ion Batteries via Vinylene Carbonate Additive and Dipropylene Glycol Methyl Ether/Water Mixed Solvent
Source: ACS Sustain Chem Eng. 2025 May 27;13(22):8363–72. doi: 10.1021/acssuschemeng.5c01897 (PMC12153231; doi:10.1021/acssuschemeng.5c01897)
Supplement: Supplementary file 1 [file sc5c01897_si_001.pdf]

Supporting Information

In Situ Modification of Sulfur-Based Cathode Electrolyte Interphases for Boosting Zinc/Graphite Dual-Ion Batteries via Vinylene Carbonate Additive and Dipropylene Glycol Methyl Ether/Water Mixed Solvent

Yitao He[a,b,], Xiaoxiang Shen[a], Jiří Červenka[b,*]*

[a] Department of New Energy Science and Engineering, School of Energy and Environment, Anhui University of Technology, Ma'anshan, China
[b] Department of Thin Films and Nanostructures, FZU – Institute of Physics of the Czech Academy of Sciences, Cukrovarnická 10/112, 162 00 Prague 6, Czech Republic

Corresponding e-mail:
* Yitao He: yitao@fzu.cz
* Jiří Červenka: cervenka@fzu.cz

Table S1. Overview of electrolyte formulations studied in this research

	Electrolyte formulation	Abbreviation
1	4 M $Zn(OTf)_2$ in H_2O:DPM(1:4)	DPMW
2	4 M $Zn(OTf)_2$ in H_2O:DPM(1:4)+1 wt% VC	DPMW-1VC
3	4 M $Zn(OTf)_2$ in H_2O:DPM(1:4)+5 wt% VC	DPMW-5VC
4	4 M $Zn(OTf)_2$ in H_2O:DPM(1:4)+10 wt% VC	DPMW-10VC

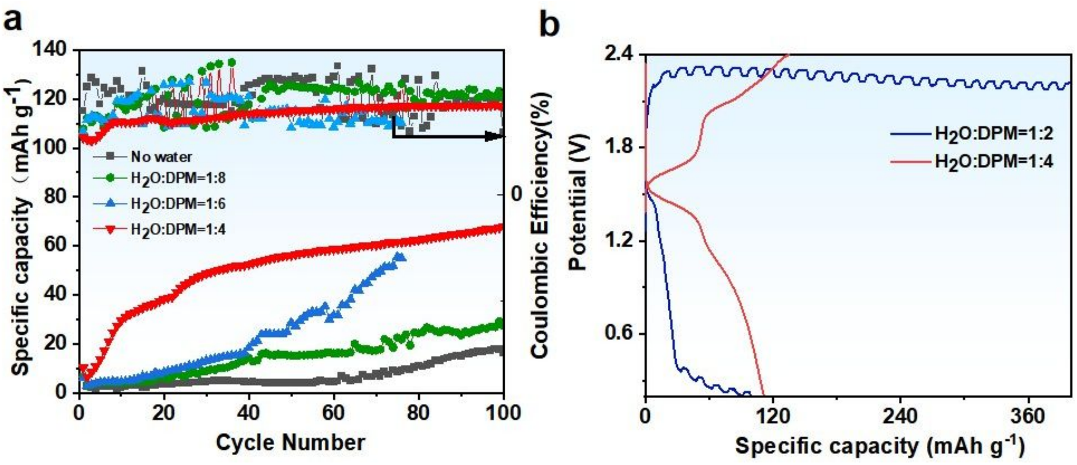

Figure S1. (a) The impact of different water-to-DPM ratios on battery performance was investigated. Five types of electrolytes were prepared, specifically: no water, H_2O: DPM=1:8, H_2O: DPM=1:6, H_2O: DPM=1:4 and H_2O: DPM=1:2, with 4 M $Zn(OTf)_2$ added to each electrolyte; **(b)** Charging/discharging curves of GP in H_2O: DPM=1:2 and H_2O: DPM=1:4 electrolytes.

As shown in Figure S1a, the electrolytes with no water and H_2O: DPM=1:8 exhibit low specific capacity and poor cycling stability. Although the H_2O: DPM=1:6 electrolyte shows higher specific capacity, it experiences significant fluctuations, and its coulombic efficiency is not very stable. In contrast, the H_2O: DPM=1:4 electrolyte demonstrates the best cycling stability and high specific capacity, while maintaining stable coulombic efficiency. As shown in Figure S1b, the electrolyte system exhibits excellent voltage tolerance at H_2O: DPM = 1:4, enabling stable charging up to 2.4 V. Further increasing the water content to H_2O: DPM=1:2 results in poor voltage tolerance, preventing the charging voltage from increasing effectively. Therefore, the H_2O: DPM=1:4 electrolyte was selected as the optimal formulation.

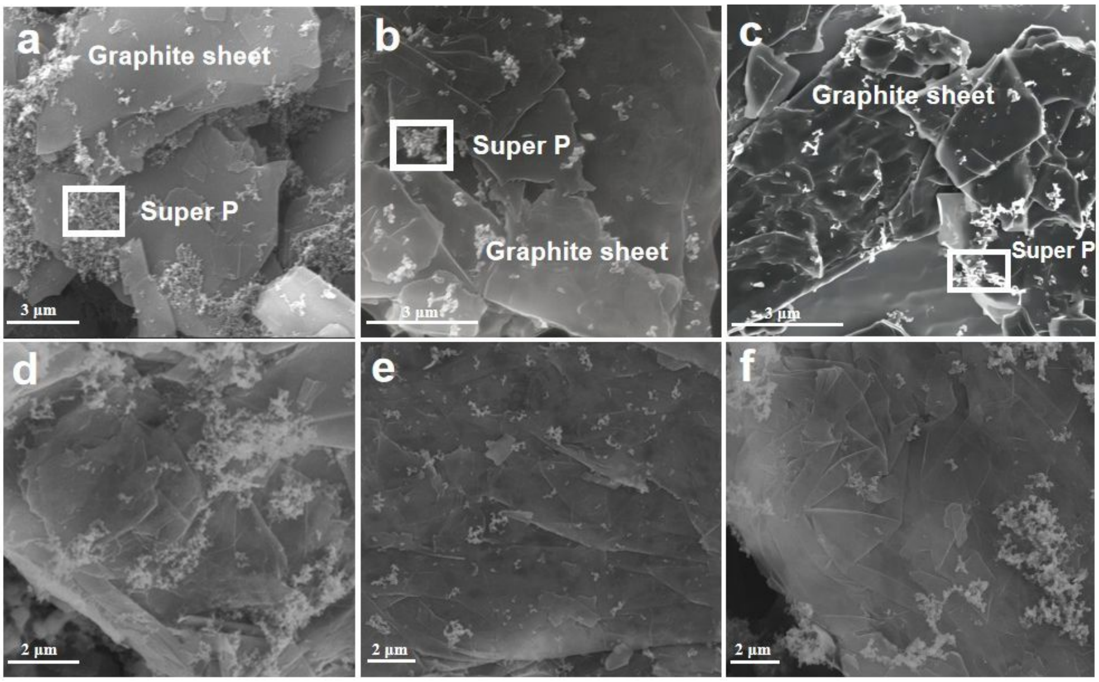

Figure S2. (a) SEM image of the original GP; **(b)** SEM image of the GP-50 in DPMW-5VC; **(c-f)** SEM image of the GP-50 in DPMW.

Compared to the original graphite, the surface of the graphite with 5 wt% VC maintains its structure and integrity, showing no cracks, corrosion, or collapse. In contrast, the surface of the graphite without VC shows minor crack damage compared to both the original graphite and the graphite with VC.

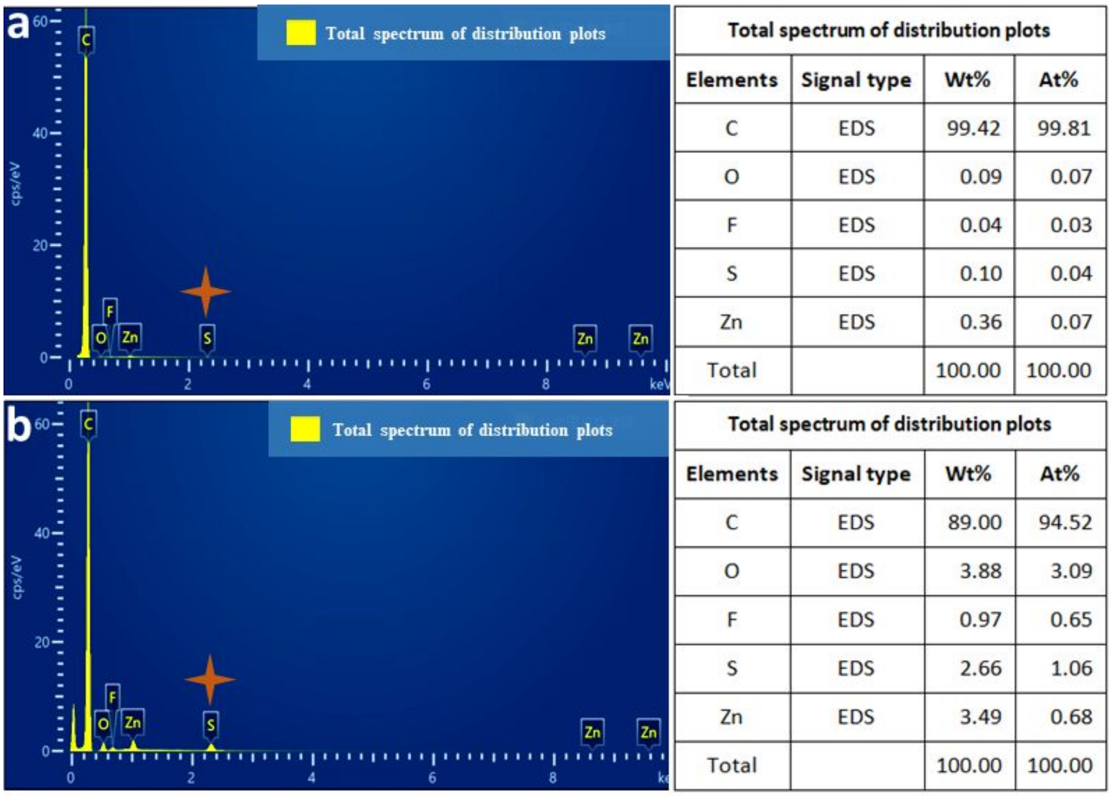

Total spectrum of distribution plots			
Elements	Signal type	Wt%	At%
C	EDS	99.42	99.81
O	EDS	0.09	0.07
F	EDS	0.04	0.03
S	EDS	0.10	0.04
Zn	EDS	0.36	0.07
Total		100.00	100.00

Total spectrum of distribution plots			
Elements	Signal type	Wt%	At%
C	EDS	89.00	94.52
O	EDS	3.88	3.09
F	EDS	0.97	0.65
S	EDS	2.66	1.06
Zn	EDS	3.49	0.68
Total		100.00	100.00

Figure S3. **(a)** EDS spectrum of the GP-50 in DPMW; **(b)** EDS spectrum of the GP-50 in DPMW-5VC. The addition of the VC electrolyte additive results in a higher sulfur content in the CEI components.

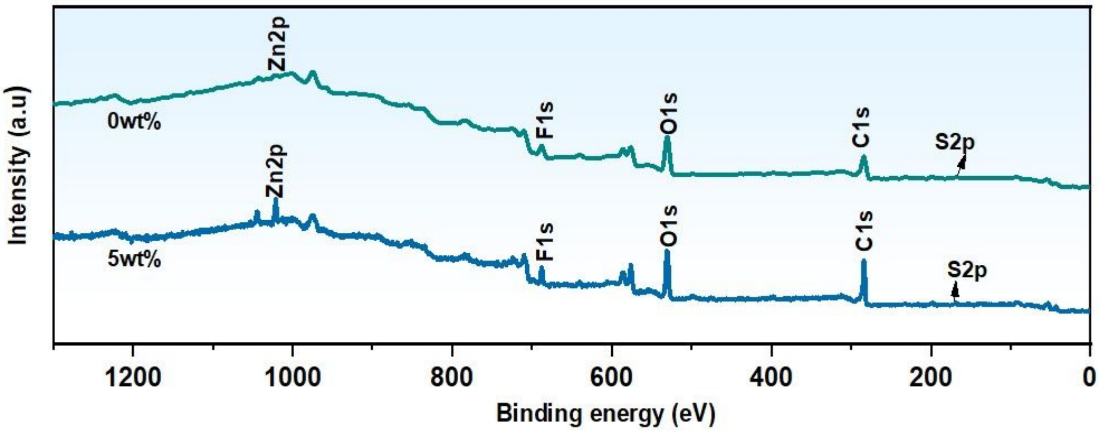

Figure S4. XPS full spectra of GP-2 in DPMW/DPMW-5VC.

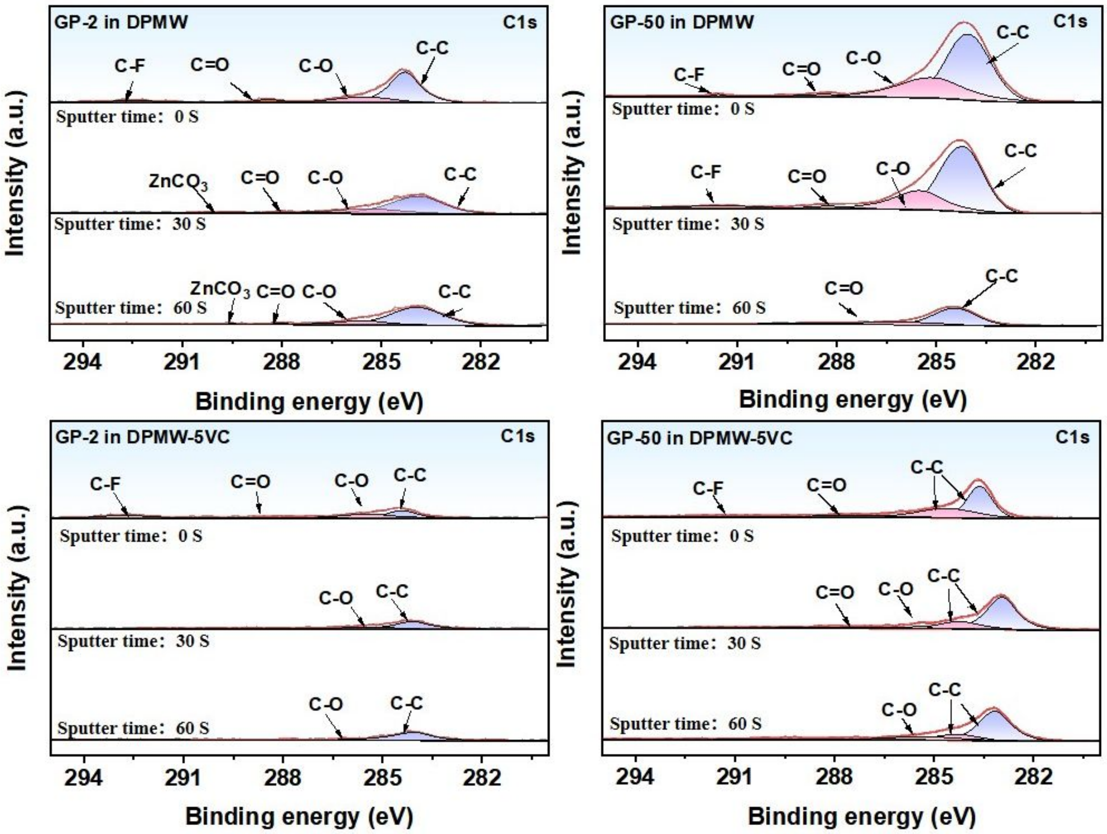

Figure S5. XPS high-resolution C1s spectra of GP-2/50 in DPMW/DPMW-5VC.

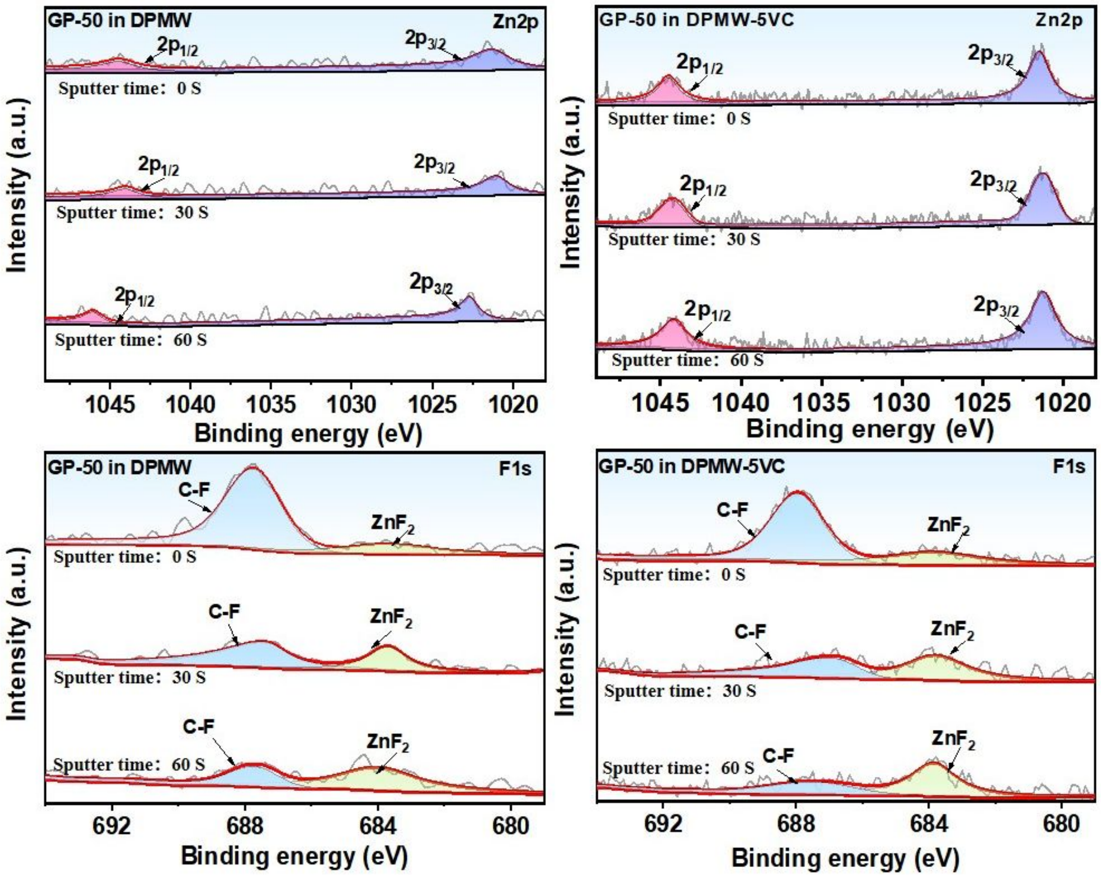

Figure S6. XPS high-resolution Zn2p and F1s spectra of GP-50 in DPMW/DPMW-5VC.

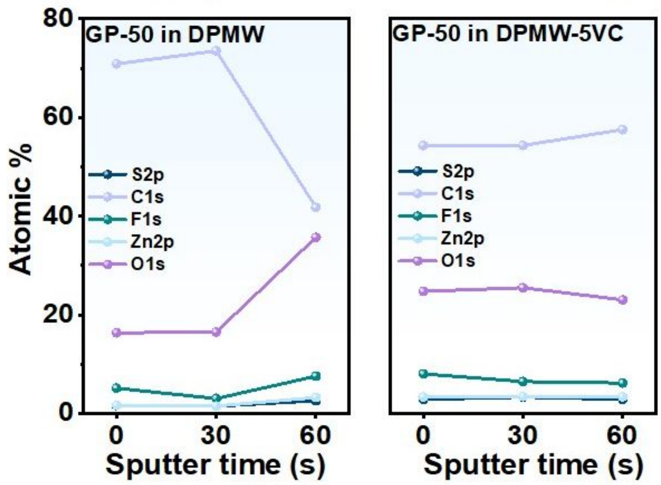

Figure S7. XPS sputtering depth profile.

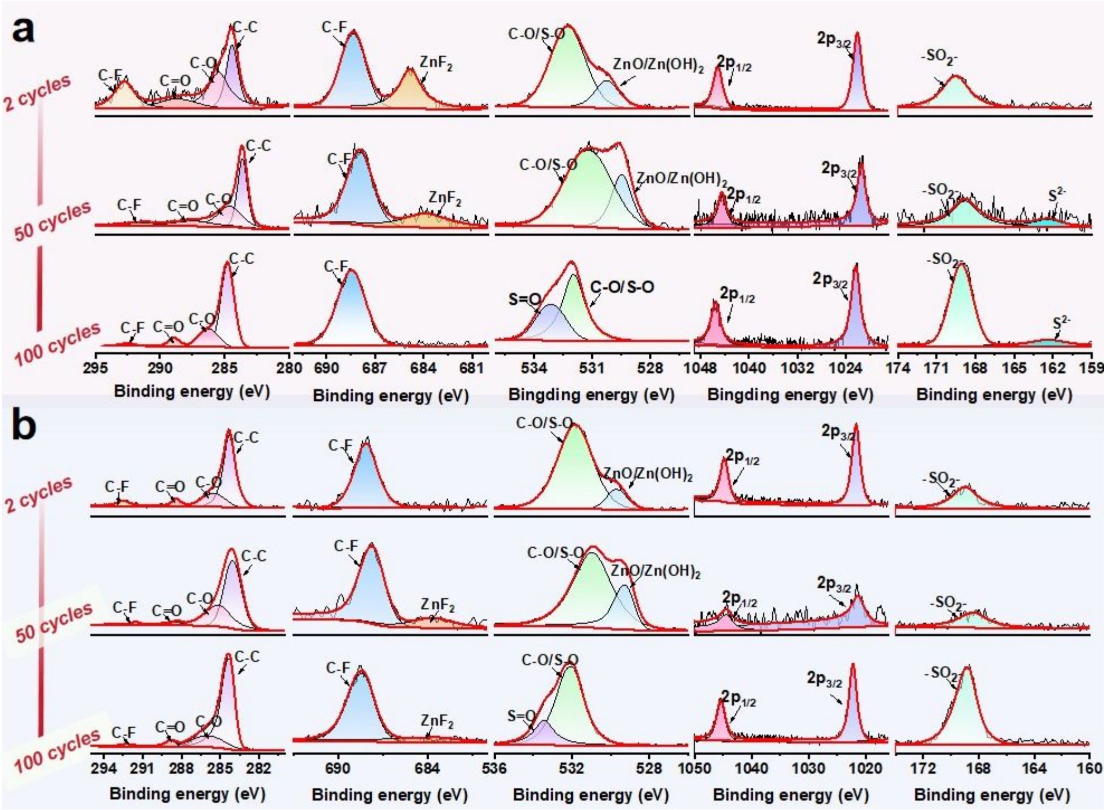

Figure S8. (a) XPS peak spectra of GP-2/50/100 in DPMW-5VC; **(b)** XPS peak spectra of GP-2/50/100 in DPMW.

To address the issue of CEI stability, we conducted XPS tests on the graphite cathode surface in DPMW-5VC and DPMW mixed electrolytes after 2 to 100 cycles to more systematically investigate the evolution of the CEI. As shown in Figure S8a, the XPS spectra of GP in DPMW-5VC electrolyte at different cycle numbers are presented. It can be seen that with the increase in cycle number, the components of the graphite CEI undergo significant changes, especially with respect to sulfur and fluorine-containing species. Specifically, after 2 cycles, the CEI formed on the graphite surface primarily consists of ZnF_2, ZnO, $Zn(OH)_2$, and $-SO_2-$. After 50 cycles, the CEI components remain similar to those after 2 cycles, dominated by ZnF_2, ZnO, $Zn(OH)_2$, but it is noteworthy that the generation of sulfides gradually becomes more apparent. After 100 cycles, the content of ZnF_2 in the CEI decreases, replaced by the appearance of S=O species, indicating an increase in sulfur content in the CEI. At the same time, the S^{2-} signal is still present, demonstrating that sulfur-containing oxides and sulfides occupy an important position in the CEI layer. In summary, with the increase in cycle number, the CEI components mainly transition from fluorides and oxides to sulfur-containing species, suggesting that sulfur-rich CEI can remain intact during long-term cycling.

Figure S8b shows the XPS spectra of GP in DPMW electrolyte at different cycle numbers. After 2 cycles, the CEI components are mainly oxides such as ZnO, $Zn(OH)_2$, and $-SO_2-$. After 50 cycles, ZnF_2 signals are detected, indicating the formation of fluorides in the surface CEI layer. After 100 cycles, the appearance of S=O signals suggests an increase in sulfur-containing oxides in the CEI. Overall, in DPMW electrolyte, the CEI components of GP primarily consist of fluorides and oxides. Overall, the main difference between the CEI of GP with and without VC is that the addition of VC promotes the appearance of sulfide components in the CEI. This can create anionic insertion sites on the graphite surface, which is crucial for improving the cycling performance of the battery. Additionally, from the in-depth analysis of XPS of GP in DPMW-5VC electrolyte from 2 to 100 cycles, it is clear that sulfur-rich CEI can remain intact during long-term cycling.

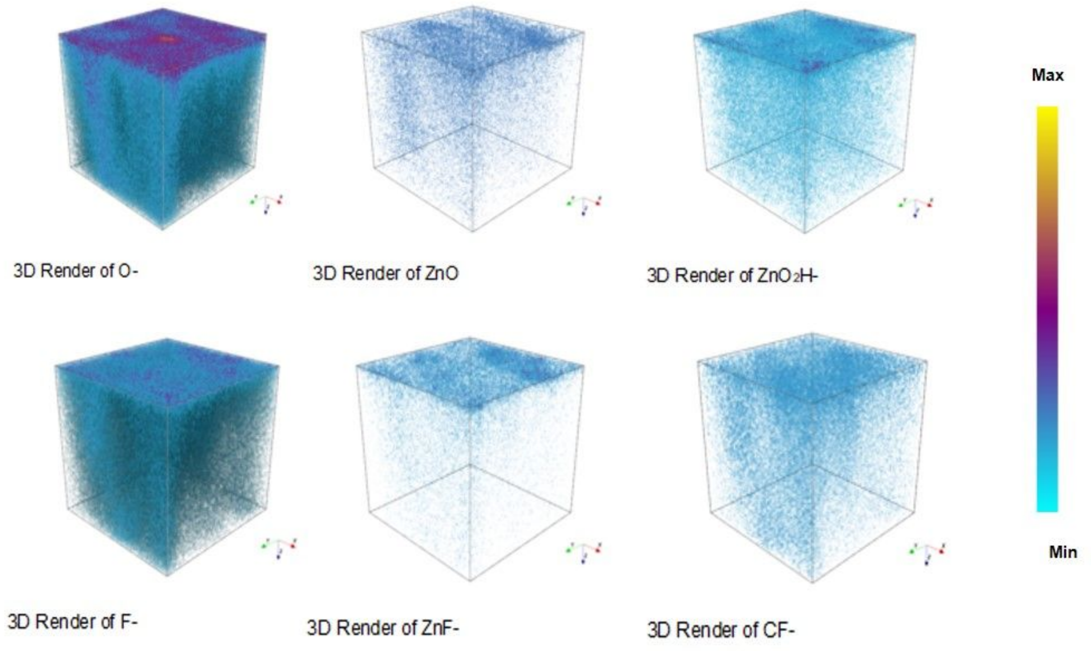

Figure S9. Depth profile of secondary ion fragments on the surface of HOPG.

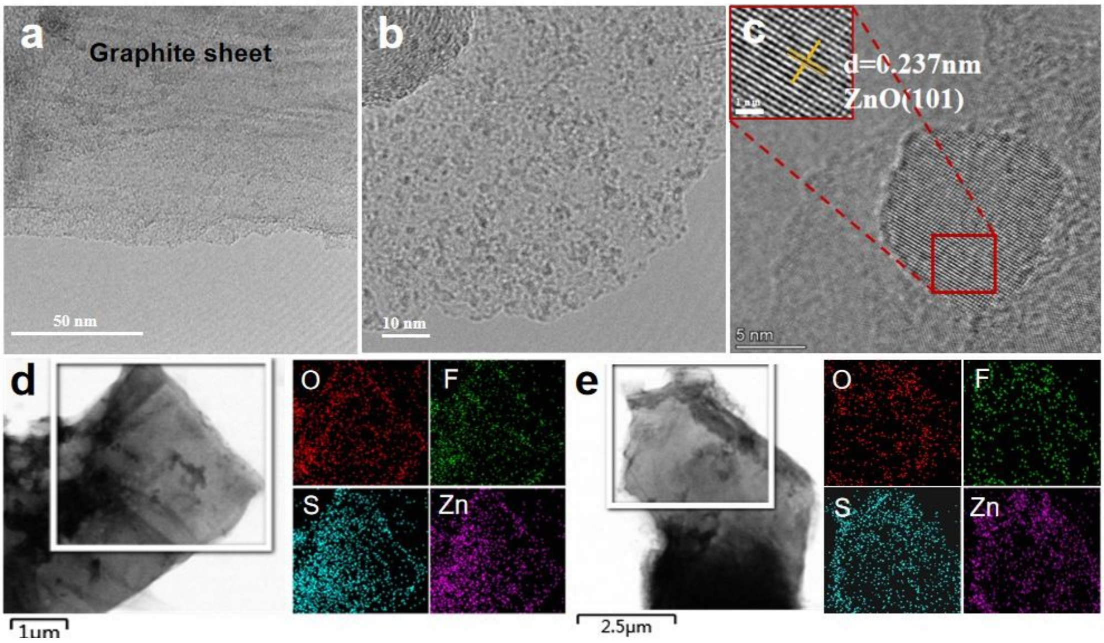

Figure S10. (a) HRTEM image of the GP-50 in DPMW; **(b, c)** HRTEM images of the GP-50 in DPMW-5VC; **(d, e)** EDX elemental mapping of the graphite cathode after cycling in DPMW-5VC electrolyte.

From these elemental maps, we can observe the distribution of different elements (O, F, S, and Zn) on the graphite cathode surface, particularly the distribution of sulfur and zinc, which provides indirect evidence for the formation of ZnS nanoparticles. However, this analysis has certain limitations, mainly because nanoparticles typically exist within the graphite lattice structure. To observe these nanoparticles on the graphite surface, the cycling process in the battery is required. During cycling, a CEI film forms on the graphite surface, which contains elements like O, S, Zn, and F. These components may interfere with the test results. Therefore, while we can broadly confirm the presence of S, accurately identifying whether it is ZnS still requires further analysis based on lattice spacing. By measuring the lattice spacing (Figure 2j) and combining the XPS and TOF-SIMS results from Figure 2 (where the S component is relatively abundant), we can hypothesize that these nanoparticles are most likely ZnS.

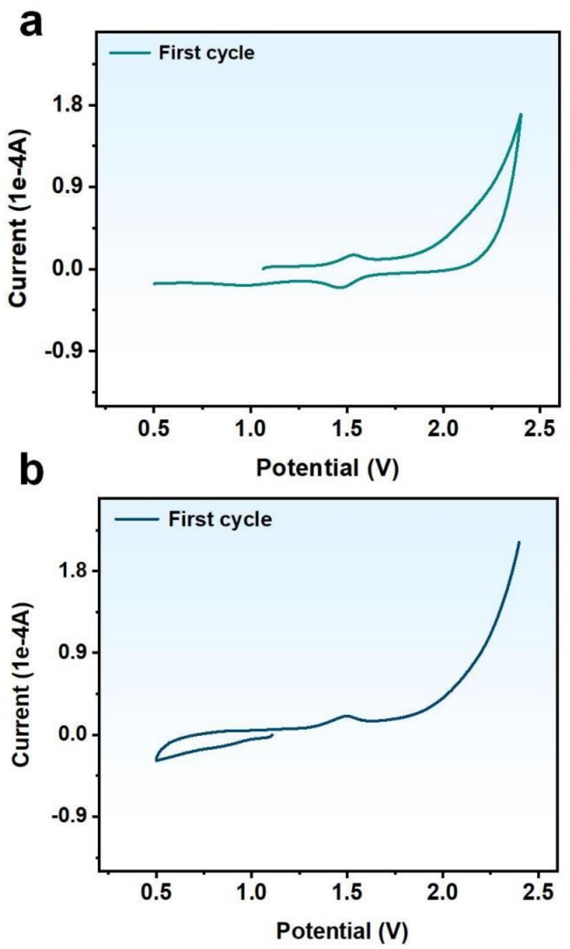

Figure S11. (a) The initial forward scan CV curve of the graphite-Zn battery in DPMW-5VC electrolyte, with a scan rate of 0.8 mV s^{-1}; **(b)** The initial reverse scan CV curve of the graphite-Zn battery in DPMW-5VC electrolyte, with a scan rate of 0.8 mV s^{-1}.

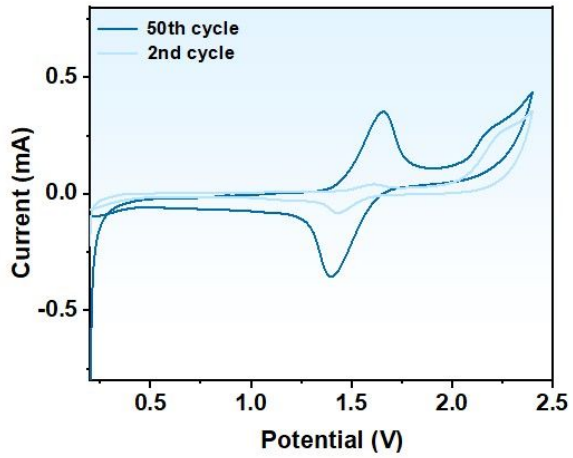

Figure S12. CV curves of the graphite-Zn battery in DPMW-5VC electrolyte after 2 cycles and after 50 cycles, with a scan rate of 0.8 mV s^{-1}.

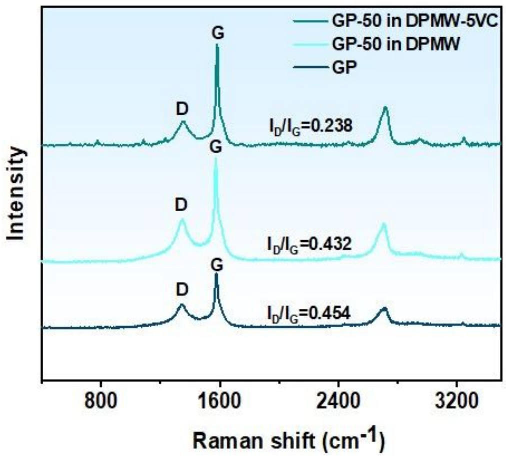

Figure S13. Raman spectra of the original GP and the GP-50 in DPMW and DPMW-5VC electrolytes.

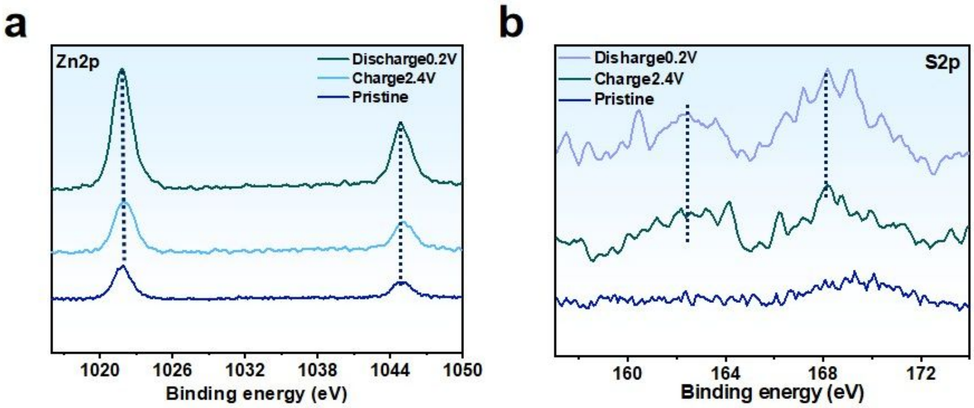

Figure S14. XPS spectra of the graphite-Zn battery in DPMW-5VC electrolyte under various charge and discharge states: **(a)** Zn2p; **(b)** S2p.

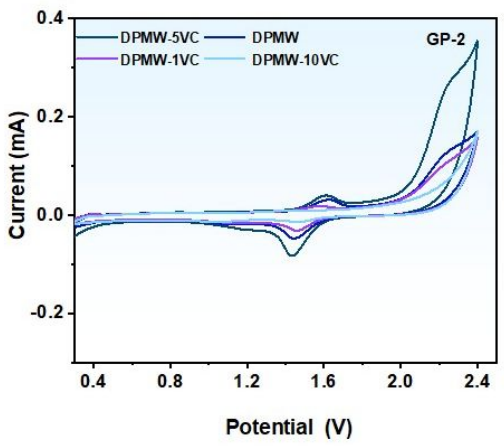

Figure S15. CV curves of the graphite-Zn battery after 2 cycles in electrolytes containing 0, 1, 5, and 10 wt% VC, with a scan rate of 0.8 mV s^{-1}.

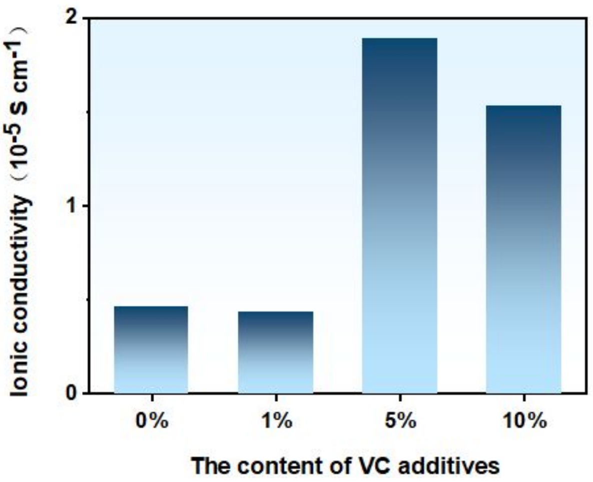

Figure S16. Calculated ionic conductivities of the graphite-Zn battery in electrolytes containing 0, 1, 5, and 10 wt% VC.

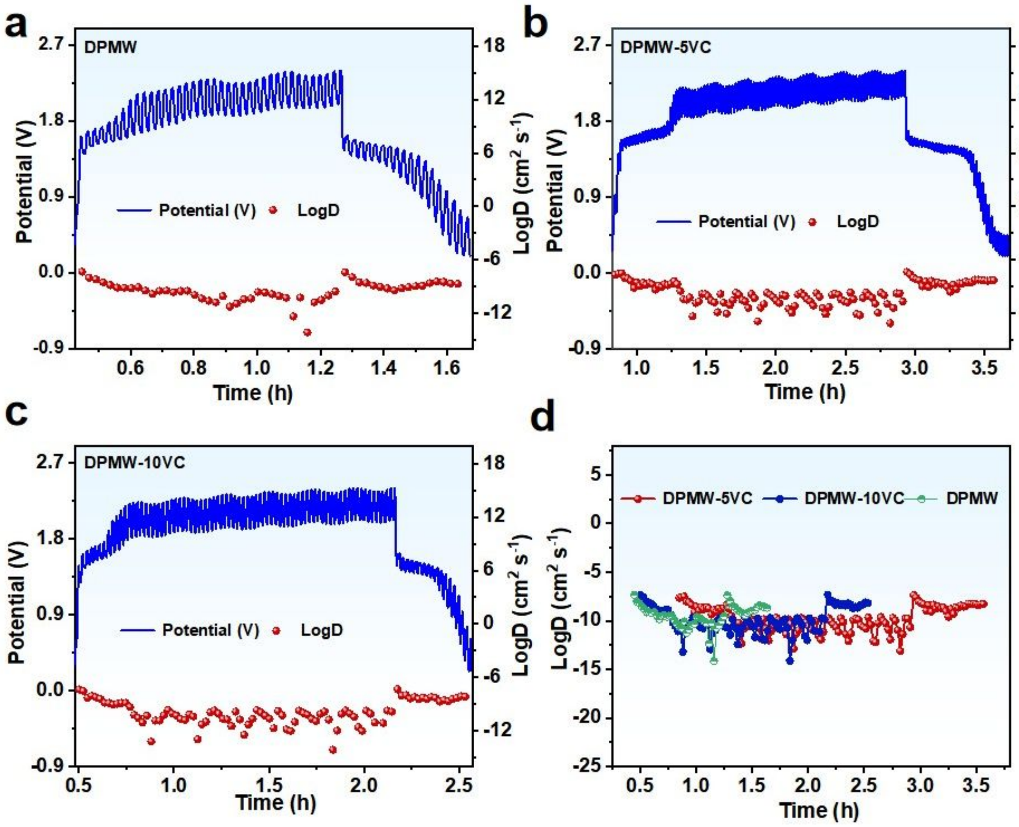

Figure S17. The GITT curves of batteries composed of **(a)** DPMW, **(b)** DPMW-5VC, **(c)** DPMW-10VC electrolytes, and **(d)** their comparison during the charge and discharge processes.

As shown in Figure S17, the GITT curves for these three electrolytes are presented. For comparison, we took the logarithm of the ionic diffusion coefficient D, and the results show that the log D value for DPMW-5VC is slightly higher, while the log D values for DPMW and DPMW-10VC are slightly lower. This indicates that although the addition of VC has some promoting effect on ionic migration, as the VC content increases (from 5 wt% to 10 wt%), its promoting effect on ionic migration diminishes, slightly reducing the ionic diffusion rate. In fact, the main role of VC is to improve the composition of the CEI and to promote the in-situ formation of sulfide nanoparticles on the graphite surface, with minimal impact on the ionic migration rate.

Table S2. Summary of calculated ionic conductivity results

Electrolyte type	d(cm)	R	A(cm^2)	σ(10^{-5} S cm^{-1})
0 wt%	0.45	4853	2.01	0.46132
1 wt%	0.45	5152	2.01	0.43455
5 wt%	0.45	1184	2.01	1.88929
10 wt%	0.45	1463	2.01	1.53028

The formula for calculating ionic conductivity (σ) is as follows:[1-3]

$$\sigma = \frac{\mathrm{d}}{AR} \qquad \text{(S1)}$$

where d is the thickness and A is the area of the cell.

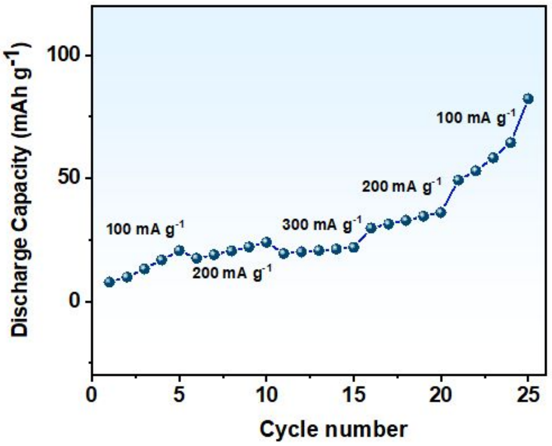

Figure S18. Rate performance of the graphite-Zn battery in 5 wt% VC electrolyte at different current densities.

Figure S18 illustrates the rate performance of the battery with 5 wt% VC. Specifically, starting at a current density of 100 mA g^{-1}, the battery's discharge capacity gradually increases with the number of cycles and stabilizes. When the current density is increased to 200 mA g^{-1} and 300 mA g^{-1}, although there is an initial drop in capacity, it quickly recovers and continues to increase in subsequent cycles. When the current density is returned to 100 mA g^{-1}, the battery not only regains its initial capacity but also exhibits a higher capacity than at the start, indicating the battery's excellent rate performance and capacity recovery ability. The reason for the observed characteristics in the rate capability curve is mainly attributed to two factors: On one hand, according to the cycling

performance test results shown in Figure 3i of the main text, it can be seen that the graphite cathode of the battery underwent an activation process within 300 cycles, with a noticeable increase in capacity, especially at the 200th cycle. Since the rate cycling was only performed for 25 cycles, the capacity increase was more significant. On the other hand, as the current density increases, the capacity tends to decrease. Therefore, these two factors together contribute to the observed features in the rate capability curve.

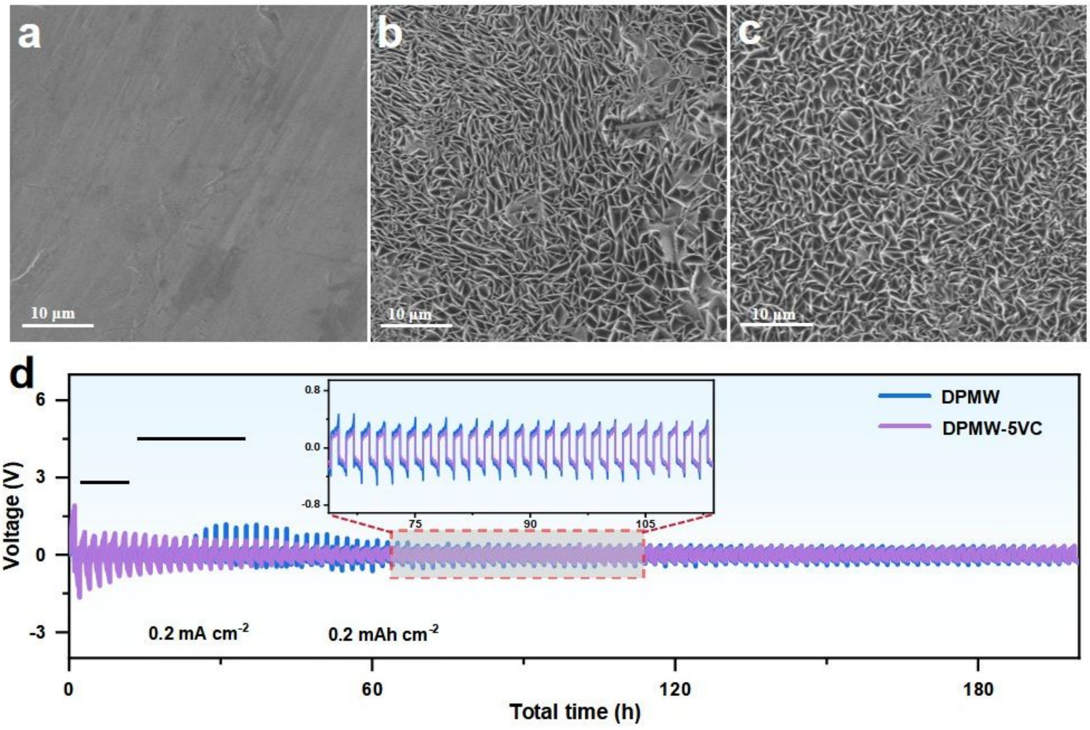

Figure S19. (a) SEM image of the surface of the original Zn foil; **(b)** SEM image of the Zn anode surface after cycling in DPMW electrolyte; **(c)** SEM image of the Zn anode surface after cycling in DPMW-5VC electrolyte; **(d)** Galvanostatic cycling performance of Zn//Zn symmetric cells in electrolytes with and without VC, with a current density of 0.2 mA·cm^{-2} and a capacity of 0.2 mAh·cm^{-2}.

As shown in Figure S19, compared to the original Zn foil (Figure S19a), although no dendrite growth is observed on the zinc surface in both cases, the surface of the zinc anode without VC exhibits uneven moss-like deposition (Figure S19b), while the surface of the zinc anode after cycling

with 5 wt% VC shows a more uniform deposition (Figure S19c). This suggests that the addition of VC helps improve the zinc deposition morphology, reduce deposition non-uniformity, and form a more uniform and dense zinc layer, thereby enhancing the stability of the zinc anode. Figure S19d shows that the voltage curves of the Zn//Zn symmetric cells with both electrolytes remain stable over 200 hours. However, the voltage curve of the electrolyte with 5 wt% VC remains stable, while the curve of the electrolyte without VC shows fluctuations between 18 to 60 hours. Additionally, the electrolyte with 5 wt% VC exhibits a smaller overpotential in the symmetric cell voltage curve. In conclusion, the addition of electrolyte additives provides some protection to the anode. However, the primary role of the electrolyte additive is reflected in improving the composition of the CEI and promoting the in-situ formation of sulfide nanoparticles on the graphite cathode surface.

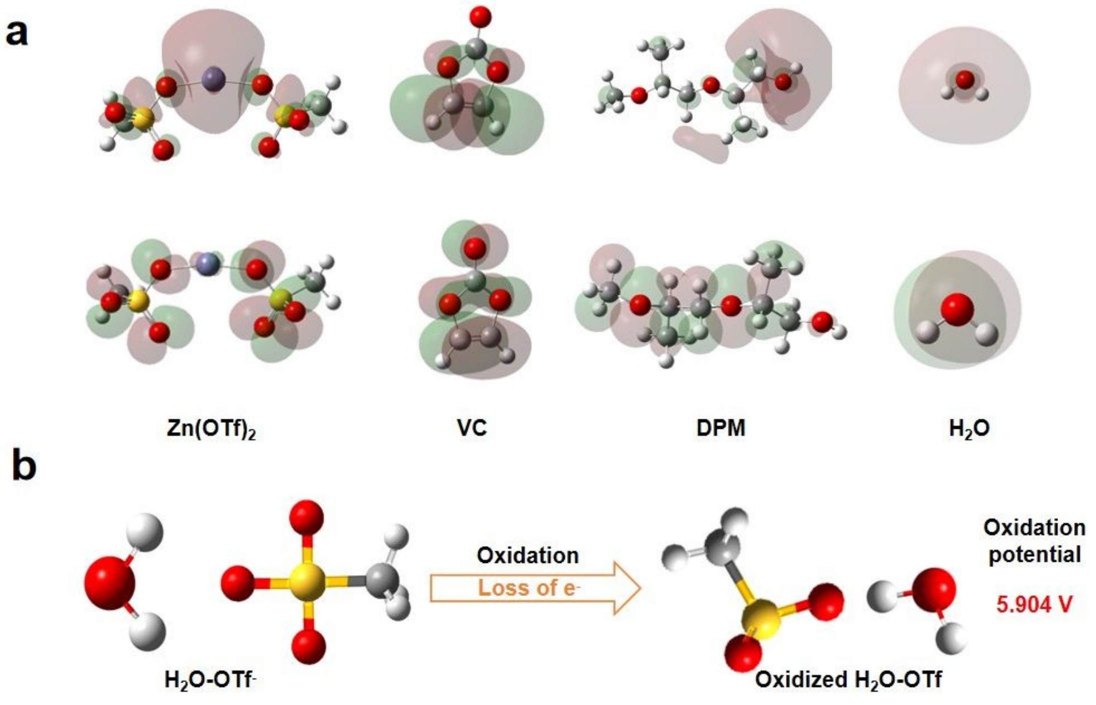

Figure S20. (a) Images of the LUMO and HOMO for electrolyte components; **(b)** Calculated oxidation potential of the H_2O-OTf^- cluster.

Table S3. Comparative electrochemical performance data of batteries from this study and previous research

Cathode	Electrolyte types	Electrolyte	Specific capacity (mAh g^{-1})	Coulomb efficiency	Cyclability (cycles)	Ref
Graphite	Aqueous electrolytes	Aqueous $Zn(ClO_4)_2$ electrolyte	45/(100 mA g^{-1})	/	500	[4]
Graphite		LiTFSI/$Zn(OTf)_2$ aqueous electrolyte	24/(200 mA g^{-1})	>95%	600	[5]
Graphite		Hybrid aqueous tetraglyme (G4) electrolyte	37	93%	500	[6]
Graphite		$MgCl_2$/Tributylmethylammonium chloride aqueous electrolyte	41	/	400	[7]
Graphite	Organic electrolytes	1.2 M $LiPF_6$ in EC/EMC	40	≈40%	50	[8]
Graphite		1.5 M $Zn(TFSI)_2$ in EMC	63.6	53.9%	100	[9]
Graphite		1 M $NaPF_6$ in EC/DMC/EMC	≈20(500 mA g^{-1})	≈80%	500	[10]
Natural graphite		1 M $LiBF_4$ in EMC/TMP	26.7(100 mA g^{-1})	/	/	[11]
Graphite	Without VC	4 M DPMW	49.44(100 mA g^{-1})	≈92%	500	This work
Graphite	Base electrolyte with additives (Other works)	$LiPF_6$/FEC electrolyte with fluorinated additive	≈60	97%	200	[8]
Graphite		1 M $Zn(TFSI)_2$/Pyr_{14}TFSI +2 wt% ES	58	>96%	500	[12]
KS6		1 M LiTFSI/Pyr_{14}TFSI+ 2 wt% ES	97(50 mA g^{-1})	≈99%	500	[13]
Natural graphite		4 M $LiPF_6$ in EMC+2 wt% VC	≈104	91%	200	[14]
Graphite	With VC	4 M DPMW+5 wt% VC	118/(100 mA g^{-1})	90%	500	This work

Table S4. Calculation of the dielectric constant of a mixed solvent

Solvent	ε_i (dielectric constant of each component)	Φ_i (volume fraction of the solvent in the mixture)	ε_{mix} (dielectric constant of the mixture)
DPM	10.5	0.72	$\varepsilon_{mix} = \sum_{i=1}^{n} \varepsilon_i \cdot \Phi_i = 33.72$ (S2)
VC	126.0[15]	0.08	
H_2O	80.4[16]	0.20	

Table S5. Calculation of binding energy

Cluster species	E(solvent with OTf^-/Zn^{2+}) (a.u.)	E(solvent) (a.u.)	E(OTf^-/Zn^{2+}) (a.u.)	E(binding energy) (eV)
DPM-OTf^-	-1166.21	-502.19	-663.92	-2.71
VC-OTf^-	-1005.30	-341.28	-663.92	-2.59
H_2O-OTf^-	-740.50	-76.47	-663.92	-2.70
DPM-Zn^{2+}	-2281.30	-502.19	-1779.09	-0.60
VC-Zn^{2+}	-2120.38	-341.28	-1779.09	-0.27
H_2O-Zn^{2+}	-1855.58	-76.472	-1779.09	-0.49

Table S6. Calculation of oxidation potential

Cluster species	G(solvent with OTf^-) (a.u.)	G(solvent with OTf^-) (a.u.)	E_{ox} (V)
DPM-OTf^-	-1165.99	-1165.73	5.57
VC-OTf^-	-1005.00	-1005.24	5.24
H_2O-OTf^-	-740.19	-740.46	5.90

References

(1) Cao, X.; Xu, S.; Zhang, Y.; Hu, X.; Yang, Y. Fluorine-doped high-performance Li_6PS_5Cl electrolyte by lithium fluoride nanoparticles for all-solid-state lithium-metal batteries. Trans. Tianjin Univ. **2024**, 30 (3), 250-261, DOI 10.1007/s12209-024-00394-1.

(2) Han, A.; Xu, S.; Wang, X.; Chang, H.; Yang, Y. Toward high-quality sulfide solid electrolytes: A liquid-phase approach featured with an interparticle coupled unification effect. Small **2023**, 20 (23), 2307997-2308010, DOI 10.1002/smll.202307997.

(3) Magalhaes, N.; Maia, B. A.; Braga, M. H.; Santos, R. M.; Correia, N.; Cunha, E. Glass fiber reinforced epoxy-amine thermosets and solvate IL: towards new composite polymer electrolytes for lithium battery applications. Int. J. Mol. Sci. **2023**, 24 (13), 10703-10715, DOI 10.3390/ijms241310703.

(4) Zafar, Z. A.; Abbas, G.; Knizek, K.; Silhavik, M.; Cervenka, J. Chaotropic anion based "water-in-salt" electrolyte realizes a high voltage Zn-graphite dual-ion battery. J. Mater. Chem. A **2022**, 10 (4), 2064-2074, DOI 10.1039/d1ta10122f.

(5) Zhang, H.; Liu, X.; Qin, B.; Passerini, S. Electrochemical intercalation of anions in graphite for high-voltage aqueous zinc battery. J. Power Sources **2020**, 449, 227594-227601, DOI 10.1016/j.jpowsour.2019.227594.

(6) Yang, D.; Watanabe, M.; Ishihara, T. Hybridizing tetraglyme to aqueous electrolyte with concentrated salts promote intercalation of anions on graphite cathode in dual-ion battery. Small Methods **2023**, 7, 2300249-2300248, DOI 10.1002/smtd.202300249.

(7) Kim, K.; Tang, L.; Muratli, J. M.; Fang, C.; Ji, X. A graphite-PTCDI aqueous dual-ion battery. ChemSusChem **2022**, 15 (5), 2394-2401, DOI 10.1002/cssc.202102394.

(8) Read, J. A.; Cresce, A. V.; Ervin, M. H.; Xu, K. Dual-graphite chemistry enabled by a high voltage electrolyte. Energy Environ. Sci. **2014**, 7 (2), 617-620, DOI 10.1039/c3ee43333a.

(9) Chen, Z.; Tang, Y.; Du, X.; Chen, B.; Cui, G. Anion solvation reconfiguration enables high-voltage carbonate electrolytes for stable Zn/graphite cells. Angew. Chem. Int. Ed. **2020**, 59 (48), 21769-21777, DOI 10.1002/anie.202010423.

(10) Yu, D.; Zhu, Q.; Cheng, L.; Dong, S.; Yang, N. Anion solvation regulation enables long cycle stability of graphite cathodes. ACS Energy Lett. **2021**, 6 (3), 949-958, DOI 10.1021/acsenergylett.1c00043.

(11) Zhang, L.; Li, J.; Huang, Y.; Zhu, D.; Wang, H. Synergetic effect of ethyl methyl carbonate and trimethyl phosphate on BF_4^- intercalation into a graphite electrode. Langmuir **2019**, 35 (11), 3972-3979, DOI 10.1021/acs.langmuir.9b00262.

(12) Ji, B.; Yao, W.; Tang, Y. High-performance rechargeable zinc-based dual-ion batteries. Sustainable Energy Fuels **2020**, 4 (1), 101-107, DOI 10.1039/c9se00744j.

(13) Rothermel, S.; Meister, P.; Schmuelling, G.; Fromm, O.; Placke, T. Dual-graphite cells based on the reversible intercalation of bis(trifluoromethanesulfonyl)imide anions from an ionic liquid electrolyte. Energy Environ. Sci. **2014**, 7 (10), 3412-3423, DOI 10.1039/c4ee01873g.

(14) Zhang, X.; Tang, Y.; Zhang, F.; Lee, C. A novel aluminum-graphite dual-ion battery. Adv. Energy Mater. **2016**, 6 (11), 1502588-1502593, DOI 10.1002/aenm.201502588.

(15) Väli, R.; Jänes, A.; Lust, E. Vinylene carbonate as co-solvent for low-temperature mixed electrolyte based supercapacitors. J. Electrochem. Soc. **2016**, 163 (6), A851-A857, DOI 10.1149/2.0541606jes.

(16) Zheng, X.; Yuan, F.; Ma, A.; Tian, S. Experimental study on microwave drying aluminum hydroxide. Coatings **2024**, 14 (6), 687-700, DOI 10.3390/coatings14060687.
